# Using UV and FTIR spectroscopy for discrimination among *vicia* seeds with emphasis on UV based multivariate modelling

**DOI:** 10.1038/s41598-025-17113-y

**Published:** 2025-09-23

**Authors:** Mai M. Ahmed, Abd El Raheim M. Donia, Yassin Ismail, Fadia S. Youssef, Sherweit H. El-Ahmady

**Affiliations:** 1 Department of Environmental Medical Sciences, Faculty of Graduate Studies and Environmental Research, Ain Shams University, Cairo, Egypt; 2 Natural Products Unit, Department of Medicinal and Aromatic Plants, Desert Research Center, Cairo, Egypt; 3 Department of Pharmacognosy, Faculty of Pharmacy, Ain Shams University, Cairo, Egypt

**Keywords:** Fava bean, *Vicia*, Discrimination, UV, *FT-IR*, Multivariate, Plant sciences, Chemistry

## Abstract

Legume Seeds of *Vicia* are cultivated and consumed worldwide for their nutritional value and bioactive compounds. Notably, *Vicia faba* (fava bean) seeds, with their many cultivars or varieties, are deeply rooted in cuisines of the Middle East and across the globe. In this work, simple and fast spectroscopic techniques, including UV and FT-IR spectroscopy, were used in combination with multivariate statistical techniques not only to discriminate among different varieties of fava beans but also to distinguish them from other *Vicia* legumes, such as *Vicia sativa* and *Vicia monantha*. In addition, the total phytochemical phenolics and flavonoids, and in vitro radical scavenging activity were assessed. Preliminary exploratory data analysis using PCA on both UV and FT-IR spectra was capable of distinguishing the seeds of fava bean varieties from other *Vicia* species. On the other hand, the FT-IR was limited in distinguishing between the varieties of fava beans compared to the UV spectra. Therefore, UV spectra were subjected to unsupervised techniques, PCA and HCA, and supervised classification techniques, SIMCA and PLS-DA, to construct useful discrimination models for eight varieties of fava beans. PCA and HCA successfully segregated the eight fava bean varieties into three informative clusters: the first cluster for the five traditional commercial Egyptian varieties, the second cluster for the two new Egyptian varieties Maryout 2 and 3, and the third cluster for the Spanish variety Luz de Otoño. Furthermore, SIMCA and PLS-DA models demonstrated good separation among these three classes of fava beans, with 100% classification accuracy for the validation set samples. In addition, the varieties of fava beans and other *Vicia* species showed a diverse content of Phenolics, flavonoids, and radical scavenging capacity, with the traditional Egyptian varieties of Sakha4 and Giza 843, as well as *Vicia sativa* and *Vicia monantha*, being the best. In conclusion, for the first time, UV spectroscopy combined with multivariate techniques could serve as a simple and fast method to distinguish between some *Vicia* seeds. Additionally, *Vicia sativa*, *Vicia monantha*, and the fava bean varieties Sakha 4 and Giza 843 might be superior to others in developing functional foods and phytopharmaceuticals.

**Supplementary Information:**

The online version contains supplementary material available at 10.1038/s41598-025-17113-y.

## Introduction

 Legumes are an important food source and play a crucial role in traditional diets worldwide^1^. Legume seeds are a rich source of essential nutrients, including proteins, carbohydrates, dietary fiber, fatty acids, vitamins, and minerals. Additionally, they contain several non-nutrient bioactive phytochemicals, such as phenolic acids, flavonoids, and condensed tannins (proanthocyanidins), which possess antioxidant properties^2–4^. The legume family exhibits remarkable diversity, encompassing over 700 genera and 19,000 species, making it the world’s third-largest flowering plant family after Orchids and Asteraceae. Within this family, the genus *Vicia*, commonly referred to as “vetches,” is the largest one within the Fabeae tribe, comprising approximately 160 species and includes a number of important food and forage crops^5^. The genus *Vicia* serves as a significant protein source for both humans and animals globally, owing to its nutritional value^2,6^.

The genus *Vicia* has gained significant popularity primarily due to its most well-known cultivated species, *Vicia faba* L., commonly known as “the fava (*faba*) bean, broad bean, horse bean, field bean”. This legume, one of the world’s oldest cultivated plants, has been cultivated since 3000 B.C. in Ancient Egypt^7^. In today’s world, fava beans are cultivated globally, including the Middle East, Europe, Latin America, and Southeast Asia. It ranks as the sixth most produced legume worldwide, with global production exceeding six million tons. While the global importance of fava bean as a widely cultivated crop and food source is well recognized, the dataset in the present study focuses primarily on Egyptian varieties of fava bean and includes only one Spanish variety. In Egypt, fava beans rank as the third most important pulse crop in terms of total production^4,8^. If Egyptian food culture were represented by a single crop, it would most likely be the fava bean. Fava bean, also known as “fūl”, has been a staple food in the Egyptian diet for centuries. It is a mainstay of Egyptian breakfast, which includes broad bean cakes “Falafel or Taamia” or stewed broad beans “Fūl medames”. It can also be consumed as a green vegetable, freshly canned, or cooked in various dishes, such as stewed broad bean paste or purée “Bissara”, and germinated broad bean soup “Fūl nabit”. Egyptians of all socioeconomic backgrounds consume an average of 6.33 Kg of fava beans per capita per year^9–11^. Moreover, recent research highlights the nutritional value of fava bean seeds as a significant source of protein (20–41%), carbohydrates (55–68%), lipids (1.2-4%), dietary fiber (12%), vitamins, and minerals. These nutritional properties have earned fava bean the reputation as “the meat of the poor”^2,3,10,12^. In addition, fava beans provide a significant amount of energy (344 Kcal/100 g)^13^.

Among the grain legumes of the *Vicia* genus that have emerged to play important roles not only as animal feed but also for human consumption are *Vicia sativa* and *Vicia monantha*. Common vetch, or *Vicia sativa*, is widely cultivated in some parts of the world and serves as both livestock fodder and a low-cost alternative for lentils in human diet^14^. *Vicia monantha*, commonly known as bard vetch, was previously referred to as black lentil in Spain. It is, however, regarded to taste inferior to lentils and was rare available in marketplaces^15^. Moreover, in Mediterranean countries, both *Vicia sativa* and *Vicia monantha* seeds or flour are utilized in soups and bread^6^.

While pulse seeds are widely recognized for their nutritional value, recent research has highlighted their abundance of phytosecondary metabolites and their potential pharmacological properties. In this regard, numerous studies have documented the diverse biological activities of various *Vicia* species, particularly fava beans, including antimicrobial, antioxidant, antidiabetic, anti-inflammatory, antidiabetic, cytotoxic and anti-Parkinson effects. Previous research has suggested the utility of fava beans as a functional food in the management of diabetes^16^. In the realm of neurodegenerative diseases, fava bean seeds and sprouts contain aromatic amino acids and L-DOPA, making them natural precursors of dopamine^8,17^. The Egyptian fava bean cultivar “Sakha 3” have demonstrated anti-Parkinsonian effects in rotenone-induced Parkinsonian mice^18^. Furthermore, fava bean seeds exhibited anticholinesterase activity against butyrylcholinesterase and acetylcholinesterase enzymes in in vitro assays, suggesting their potential utility in the management of mild or early-stage Alzheimer’s disease^19^. In addition, some in vitro studies showed the antioxidant properties of *Vicia sativa*, *Vicia monantha* and many cultivars and genotypes of fava bean^2,8,20–22^. Various *Vicia* species have been investigated for the anti-inflammatory and antinociceptive properties in numerous in vivo models. For instance, ethanolic extracts derived from the aerial parts of *Vicia sativa* have demonstrated inhibitory effects against various inflammatory and nociceptive mediators^23^. In a rat model of the ulcerative colitis condition, dietary supplementation with fava bean substantially improved the impaired oxidative stress and inflammatory biomarkers associated with this condition^24^. Regarding the cytotoxic and anticancer activities of the *Vicia* species, a limited number of studies have successfully isolated various phytochemicals, including fatty acids, triterpenes, flavonoids, and coumarins, from *Vicia sativa* and *Vicia monantha* that demonstrated promising anticancer activity against a bunch of cell lines^25–27^. Similarly, extracts derived from three Australian fava bean varieties decreased the proliferation of some human cancer cell lines^28^.

Fava bean cultivars or varieties have been adapted to suit specific local environments^29^. Significant genetic variation has been documented in fava bean varieties, particularly in morphological traits like seed size and chemical composition^2,30^. In addition to genetic differences, environmental factors, such as soil composition, climatic conditions, and culture, significantly influence the variation in chemical profile and overall quality among fava bean cultivars^2,12^. Moreover, different cultivars may be mixed intentionally or unintentionally through commercial adulteration to combine desired traits or maximize economic return^29^. Given the variation in yield, nutritional quality, antinutritional factors, and bioactive phytochemicals among cultivars and species, there is a need for efficient, rapid, low-cost and objective evaluation methods to discriminate and classify fava bean varieties. Furthermore, to conserve the valuable genetic diversity within the genus, particularly for endemic species, it is essential to accurately and effectively discriminate between closely related *Vicia* species^31^. This enables seed authentication and helps avoid adulteration, supports agronomic decision-making, facilitates the development of functional foods or phytopharmaceuticals, and aids in the conservation of traditional and endemic genetic resources, highlighting its importance for food industry and safety, crop improvement, phytopharmaceuticals development and biodiversity preservation. One direction of research exploited the heterogeneity of phenotypic morphological characters, such as seed size, to discriminate between some fava bean cultivars^29^. Another direction of research used genetic approaches to study the diversity within fava bean varieties as well as some closely related *Vicia* species^31,32^. Furthermore, the variability among fava bean varieties and the complexity of their phytochemical compositions inspired further research to differentiate between these varieties based on their bioactive components and biological properties^13^. For example, some studies assessed the differences in the polyphenols and condensed tannins in their local varieties and demonstrated a significant variation in the concentrations of these compounds which affect the quality of these varieties^2,12,21^. Moreover, a few studies have employed advanced techniques, such as hyphenated tandem mass spectrometry to assess the differences between the metabolite profiles of some varieties of fava bean or some species within the genus *Vicia*^4,8,33,34^. Given the recent interest in utilizing simpler and faster techniques, such as UV and IR spectroscopy, to differentiate between varieties within a species or between different species, our study sought to employ spectroscopic techniques to discriminate not only between fava bean and other *Vicia* species but also between various fava bean cultivars.

Recently, many analytical techniques, including chromatographic fingerprinting, hyphenated tandem mass spectrometry, nuclear magnetic resonance, and various spectroscopic techniques coupled with multivariate statistical methods, have been recognized as essential tools for evaluating the chemical profiles of diverse food and plant materials, as well as for discriminating between these substances. However, many of these analytical techniques require expensive equipment, incur high operational costs, and involve lengthy data acquisition and analysis processes. In contrast, simple and rapid spectroscopic techniques, such as ultraviolet and Fourier transform infrared spectroscopy, have been adopted as effective approaches for the identification and discrimination of medicinal plants, pharmaceuticals, and food^35–40^. It has been reported that UV and infrared spectroscopy, coupled with multivariate statistical models, provide a reliable and efficient alternative for differentiating food systems. UV spectroscopy acted as an effective discriminatory tool for authenticating green tea^41^as well as various Thyme and Curcuma samples^42,43^. Moreover, it was very useful in discrimination and classification of coffee varieties, tea varieties, and sauces^44–46^. Vibrational spectroscopy, particularly infrared spectroscopy, is a widely employed fingerprinting technique for authenticating food products and medicinal plants, particularly when coupled with chemometric analysis^47^. This technique is non-destructive, rapid, accurate, requires minimal sample amounts, and does not require reagents, making it eco-friendly^48^. Fourier transform infrared spectroscopy successfully discriminated between various moss species based on their unique spectral profiles, in conjunction with chemometric analysis^49^. Furthermore, several studies reported that coffee and tea varieties with different quality were discriminated based on their FT-IR spectra combined with different statistical methods^50,51^. Regarding the discrimination between *Vicia* species, a single study has employed liquid chromatography-tandem mass spectrometry, in conjunction with multivariate statistical chemometric methods, to differentiate between 16 *Vicia* species^4^. Nevertheless, chemical analysis methods such as liquid chromatography-tandem mass spectrometry are complex and time-consuming, making them unsuitable for rapid discrimination between *Vicia* species and fava bean varieties. Therefore, there is a need to develop faster, simpler, and more cost-effective methods based on simple spectroscopic techniques, such as UV and infrared spectroscopy, for rapid discrimination between different *Vicia* species and varieties. To our knowledge, no previous studies have applied UV spectroscopy to discriminate between the seeds of *Vicia* species and fava bean varieties or cultivars. In addition, only a few studies have utilized infrared spectroscopy in the mid- or near-infrared regions for the qualitative discrimination of different fava bean samples based on their cultivars (genotypes), geographic origin, seasonal variations, and their nutrient or bioactive compound content^52^.

The primary objective of this study was to develop a proof-of-concept framework and assess the feasibility of UV and ATR-FTIR spectroscopy, coupled with multivariate analysis, to discriminate between *Vicia faba* samples and other *Vicia* seeds, including *Vicia sativa* and *Vicia monantha*, as well as among the different *Vicia faba* cultivars or varieties. The simplest, most appropriate, and most efficient method was used to build and validate discrimination and classification models for the traditional Egyptian fava bean varieties, the new Egyptian varieties Maryout 2 and 3, and the Spanish variety Luz de Otoño. Moreover, the comparison of the total phenolic content, flavonoid content, and DPPH antioxidant capacity of these important legumes was conducted.

## Materials and methods

### Plant material

The study included 8 fava bean varieties sourced from Egypt, representing those accessible to us, as well as 2 other *Vicia* species. A total of 67 samples of dried seeds of *Vicia* species (*V. monantha*, *V. sativa*, and eight cultivars or varieties of *V. faba*, namely, Sakha 1, Sakha 4, Giza 716, Giza 843, Maryout 2, Maryout 3, Masr 1, and Luz de Otoño) were used for the present study. The traditional commercial Egyptian *Vicia faba* cultivars were represented by Sakha 1, Sakha 4, Giza 716, Giza 843, and Masr 1, while Maryout 2 and Maryout 3 represent two new Egyptian cultivars. The Luz de Otoño *variety* is a Spanish variety of fava bean imported into Egypt by the Unifert Misr-Samtrade agricultural company (Cairo, Egypt). Table 1 Contain the codes for the eight fava beans varieties, *Vicia sativa*, and *Vicia monantha*. The agricultural Research Centre in Giza, Egypt, provided and identified the dried seeds of the above Egyptian cultivars of fava beans in March and April of 2022, except for Maryout 2 and Maryout 3, which were provided and identified by Prof. Dr. Sayed Abdel Salam Hassan Omar, Professor of Plant Breeding, Desert Research Center, Cairo, Egypt. *V. monantha* and *V. sativa* were collected from their natural wild habitats in Sidi Barrani, Mersa Matruh governorate, Northwestern Coast, Egypt, and were identified by Dr. Omran Ghaly, Head of the Plant Taxonomy Unit, Desert Research Center, Cairo, Egypt. Voucher specimens with code numbers CAIH-1256-R(1–10), respectively, were kept at the herbarium of the Desert Research Center.

**Table 1:** Codes assigned for the identification of *Vicia sativa*, *Vicia monantha* and 8 varieties of *Vicia faba.* The codes for the validation set samples of the 8 varieties of *Vicia faba* take the same code plus the letter “P”. For example, to code sample 1 from Luz de Otoño variety in the validation set, it is LUZ1P.

Plant sample	Sample code
*Vicia monantha*	VM
*Vicia sativa*	VS
*Vicia faba* (Giza 716)	GZA
*Vicia faba* (Giza 843)	GZB
*Vicia faba* (Luz de Otoño)	LUZ
*Vicia faba* (Masr 1)	MSR
*Vicia faba* (Sakha 1)	SKHA
*Vicia faba* (Sakha 4)	SKHB
*Vicia faba* (Maryout 2)	MRA
*Vicia faba* (Maryout 3)	MRB

### Sample Preparation for chemometric study

The stock solution was prepared by grinding plant seeds and macerating 2 g of each sample in 50 mL of HPLC-grade methanol for 45 min using sonication. For UV spectroscopic analysis, 1 mL of the sample solution was diluted to 10 mL with methanol. Regarding FTIR spectroscopy, a portion of each dried powdered sample was taken and ground separately using a mortar, then mixed well with potassium bromide in a ratio of 1:30, respectively, to create an intact transparent disc that was needed for exposing the sample to IR radiation.

### Ultraviolet spectroscopy (UV)

UV spectroscopic analyses were performed on all the prepared samples using a Thermo Scientific Evolution 300 UV-Vis Spectrophotometer equipped with a quartz cell that provided a 1 cm optical path and 1 nm spectral resolution over the ranges 200–400 nm for UV spectroscopy. Triplicate measurements were taken for each sample.

### Fourier transform infrared spectroscopy (FTIR)

The FTIR spectra of samples were scanned using ATR-FTIR Spectroscopy, THERMO NICLOT, 50, with the IR radiation spectrum (4000–400 cm^-1^). The measurements were taken in triplicate.

### Chemometric analysis

The multivariate methods and chemometric techniques employed in this study were carried out using the Unscrambler X 10.4 software. Full cross validation (Leave-one-out strategy) was implemented as an internal validation strategy in Unscrambler to maximize the use of data and check the multivariate model performance (PCA and PLS-DA). In addition, external validation with independent dataset (not used in model training) was applied to check the performance of classification and discrimination models on unknown samples.

#### Unsupervised techniques: principal component analysis (PCA) and hierarchical cluster analysis (HCA)

PCA was utilized as a data reduction technique to create a visual plot for qualitative assessment of the samples’ similarities and differences. The Scatter score plots of the very first few principal components (PCs) were produced to identify and assess groupings, trends, and outliers. Initially, preliminary exploratory data analysis was conducted on 10 *Vicia* samples to determine the feasibility of UV and FT-IR techniques to discriminate between the samples. The UV and IR spectral data of 10 *Vicia* samples were subjected separately to principal component analysis (PCA) and Leave-one-out-cross validation method (full cross validation) was implemented to check the model performance. After that, only the UV spectra of a total of 67 samples were utilized to train and validate unsupervised and supervised discrimination and classification models. The UV spectra of 40 samples (4 samples per variety/species) were used to construct a PCA model that includes the 8 Fava bean varieties as well as *Vicia sativa* and *Vicia monantha*. Following that, another PCA model was trained based on 32 samples of only the 8 fava bean varieties (4 samples per variety, excluding the samples of *Vicia sativa* and *Vicia monantha*). Next, the later PCA model was validated and challenged by 25 samples of fava beans (3 sample per each variety, except Luz de otoño, 4 samples were used) and 10 samples of the two other *Vicia* species (5 samples per species).The HCA applied to UV spectra to distribute the 40 samples of the fava bean varieties and the other two species into groups using the complete linkage method for cluster building, and the distance between clusters was computed by the Euclidean method.

#### Soft independent modeling of class analogy (SIMCA) classification model

SIMCA is a well-established multivariate classification methodology that relies on the PCA of each individual class. The previous training set of 32 samples representing 8 fava bean varieties (4 samples per each variety) was used to train PCA class models that describe the majority of variation in 3 classes of fava bean. The three classes include class1: the five traditional Egyptian fava bean varieties, class2: the Spanish variety Luz de otoño; and Class 3: the two new Egyptian varieties of Maryout 2 and 3. The SIMCA model was then used to predict the class of another set of 35 unknown samples (validation set), consisting of 25 samples of fava beans (3 sample per each variety except Luz de otoño, for which 4 samples were used) and 10 samples for *Vicia sativa* and *Vicia monantha* (5 samples per species). If a new sample is sufficiently similar to the others in a specific class, it is recognized as a member of that class.

#### Partial least squares discriminant analysis (PLS-DA)

PLS-DA (Partial Least Squares Discriminant Analysis) is a supervised pattern recognition method that leverages the strengths of both PLS regression and classification techniques. Building upon the PLS regression algorithm (PLS1 with one dependent Y variable, PLS2 with multiple dependent Y variables), PLS-DA identifies latent variables that exhibit maximum covariance with the Y variables. In this study, the discriminant process of PLS-DA involved the following steps: (1) each of the 32 sample of the previous training set, was assigned a dummy variable. This dummy variable served as a reference value, arbitrarily indicating whether or not the sample belonged to a specific class^53–55^. The eight fava bean varieties were organized into 3 classes representing the traditional Egyptian fava bean varieties, the Spanish variety (Luz de Otoño), and the two new Egyptian varieties (Maryout 2 and 3) and the Y categorical value for each class was encoded in two dimensions using two numbers (−1 or 1), respectively^53–55^. The first class of the traditional Egyptian fava bean varieties was encoded into “−1, − 1” for dimension one and two, respectively. The second class of the Spanish variety Luz de Otoño was encoded into “−1, + 1,” and the third class of new Egyptian varieties Maryout 2 and 3 was encoded into “+1, + 1”^53^. (2) To construct PLS models, a PLS regression was conducted between the categorical variables and the corresponding spectral data. (3) Utilizing the established PLS models, the categorical variables of 35 unknown samples (validation set) were predicted. To classify an unknown sample as a member of the first class, the predicted Y values must be less than 0, and the deviation must be less than 1. Conversely, for an unknown sample to be assigned to the third class (Maryout 2 and 3 varieties), both predicted Y values in the two dimensions must be greater than 0, with a deviation less than 1. Finally, to classify an unknown sample as a member of the second class of the Spanish variety Luz de Otoño, the predicted Y values of the first dimension must be < 0 and the second dimension must be > 0, and the deviation is < 1.

### Determination of total phenolics, flavonoids, and antioxidant capacity

To prepare methanol extracts of *Vicia* seeds, 10 g of *Vicia* seed powder were macerated in 100 ml methanol, mixed, allowed to sit overnight, and then filtered through filter paper. The filtrate was stored in a dark-glass bottle. Following that, the residue was further extracted with methanol twice and the two filtrates joined the first one. The filtrates were concentrated under reduced pressure using a rotary evaporator. The resulting extracts were collected and dried in a desiccator to a constant weight, then kept in dark-glass bottles for subsequent analysis^56^.

#### Determination of total phenolic content (TPC)

The total phenolic content of methanolic extracts from *Vicia* seeds was quantified using the Folin-Ciocalteu technique^57,58^. To determine TPC, 0.2 mL of the methanolic extract (1 mg/mL) was combined with 1 mL of Folin-Ciocalteu reagent and 0.8 mL of 7.5% sodium carbonate. The reaction mixtures were left to stand at room temperature for 60 min, after which the absorbance was measured at 765 nm using a spectrophotometer. The TPC content of different extracts was performed in triplicate. The results were expressed as mg of gallic acid equivalent (GAE) per g extract from a calibration curve.

#### Determination of total flavonoid content (TFC)

The AlCl_3_ colorimetric method was used to determine the content of flavonoid compounds in methanolic extracts of *Vicia* seeds^59,60^. In short, a 0.5 mL solution of the methanolic extract of *Vicia* seeds (1 mg/ml) were mixed separately with 1.5 mL of methanol, 0.1 mL of 10% aluminum chloride, 0.1 mL of 1 M potassium acetate, and 2.8 mL of distilled water and kept at room temperature for 30 min. The reaction mixture’s absorbance was measured at 415 nm using a spectrophotometer. The total flavonoid content was determined using a calibration curve and expressed as mg of quercetin equivalent (QE) per g extract.

#### In vitro antioxidant evaluation using the diphenyl picrylhydrazyl radicle scavenging capacity assay (DPPH^•^)

Each test sample (1 ml), containing one of these five concentrations (0.1, 0.25, 0.5, 1, 2 mg) of crude *Vicia* seed methanolic extract, was mixed with 3 ml of methanol and 1 ml of 0.1 mM DPPH solution. The mixture was thoroughly shaken and incubated in darkness at room temperature for 30 min. The control sample was composed of 4 ml of methanol and 1 ml of DPPH while 5 ml of methanol was used as a blank^61^. After 30 min under dark conditions, the absorbance of the samples was measured at a wavelength of 517 nm against the blank using a UV-Vis spectrophotometer. Three sets of measurements were taken for each parameter. The percentage of DPPH inhibition or the % radical scavenging activity (%RSA) was calculated using the following equation: (%RSA) = [(Absorbance of the control - average absorbance of the sample)/Absorbance of the control] x 100. The IC50 value represents the concentration of the sample required to inhibit 50% of the DPPH radicals. The IC50 was determined by non-linear regression graph between the percentage of radical scavenging activity (%RSA) and the concentration of the sample^62^.

## Results and discussion

### Preliminary exploratory data analysis for the discrimination of *vicia* samples using UV and FT-IR spectroscopy

**Fig. 1:**
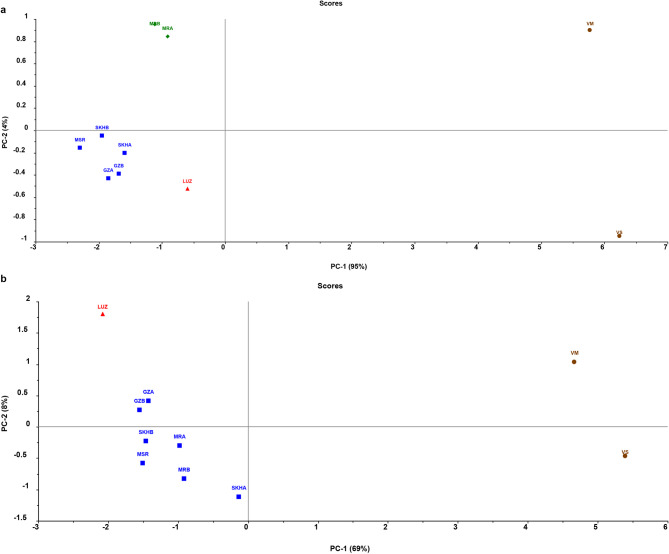
PCA score plots resulted from preliminary exploratory data analysis of **(a)** UV spectra and **(b)** IR spectra of 10 *Vicia* seeds samples.

The UV absorbance spectra of the methanol extracts of the ten *Vicia* samples were measured in the range of 200–400 nm, and the absorption bands appeared in the spectral range between 216 and 384 nm **(**Fig. 1S**).** The UV absorption bands of the different *Vicia* methanolic extracts are likely due to the existence of different UV active chromophores, such as aromatic, carbonyl, and various conjugated systems, in the *Vicia* phytochemicals that undergo π,π* and n,π* transitions^63^. In this study, the maximum UV absorbance (λmax) of *Vicia* samples was observed at 276 nm. Our observation is similar to previous couple of studies that reported UV absorbance maxima at 276 nm for fava beans crude extract, its low-molecular weight phenolic fraction, and another condensed tannin fraction^21,64^. This can be partially attributed to the abundance of complex array of phytochemicals in *Vicia* seeds, which have a UV maximum close to 276 nm and are mostly composed of phenolic acids, flavonoids, condensed tannins, alkaloids, and jasmonates^4,8,21,33,34,64–67^. The majority of secondary metabolites in *Vicia* seeds are phenolic acids and polyphenols. Most Phenolic acids of *Vicia* seeds are classified into two types: hydroxycinnamic acid derivatives and hydroxybenzoic acid derivatives. The most common hydroxycinnamic acids in *Vicia* seeds with their UV absorbance maxima (λ_max_) are the ferulic (λ_max_ 218, 236, 285, 300), coumaric (λ_max_ 226, 285, 305**)** chlorogenic, caffeic (λ_max_ 220, 240, 294, 326), sinapic (λ_max_ 238, 322) acids^4,8,33,34,65–69^. While the most prevalent hydroxybenzoic acids in *Vicia* seeds include *P*-Hydroxybenzoic acid (λ_max_ 255), protocatechuic (λ_max_ 260, 295), Protocatechuic aldehyde (λ_max_ 280,311), syringic (λ_max_ 276), vanillic (λ_max_ 261, 294), vanillin, gallic (λ_max_ 272), and salicylic (λ_max_ 231, 296**)** acids^4,8,21,33,34,65–68,70^. Fava beans and other *Vicia* seeds are rich in various flavonoid classes. The most abundant flavonols, including quercetin (λmax 255, 370), kaempferol (λmax 266, 367), myricetin (λmax 254, 374), isorhamnetin (λmax 253,370), and rutin (λmax 259, 359) as well as the flavan  -3-ols such as catechin (λmax 279) and epicatechin (λmax 279) and their gallate derivatives (λmax 274)^4,6,8,20,21,33,34,64–67^. The flavones, including apigenin (λmax 267, 296, 336), luteolin (λmax 253,267,349), naringenin (λmax 289, 326), and vitexin (λmax 270, 335) are also present in *Vicia* seeds. Furthermore, the isoflavones, such as genistein (λmax 261) and daidzein (λmax 249, 303) have been reported to be found in *Vicia* seeds in a lesser amount. Moreover, chalcones, such as isoliquritigenin (λmax 258, 298, 367) and phloretin have been isolated or detected in *Vicia* species^4,6,8,20,33,34,65–67,71,72^. In addition, *Vicia* seeds are also a significant source of polyphenolic compounds, notably condensed tannins (proanthocyanidins) such as procyanidin and prodelphenidin and their derivatives with λmax of 276–279 ^4,8,21,64–66^. Among the major nitrogenous compounds that have been reported in *Vicia* seeds are vicine and convicine, the chief alkaloids in *Vicia* seeds, with λmax 275 and 271 respectively^4,6,8,73^. In addition, *Vicia* seeds contain many nutritive amino acids, such as tryptophan, tyrosine, and phenylalanine, among others, and their bioactive derivatives, such as L-dopa, which may elicit UV absorption features in the range of 257–280 nm^17,74^. Regarding the jasmonate class, a handful of phytochemicals have been identified in *Vicia* seeds, including jasmonic acid, Wyerone, wyerone epoxide, tuberonic acid, and ethyl jasmonate with λmax around 220 and 290 ^4,8,67,75-77^.

Preliminary exploratory data analysis was performed on the average absorbance of three replicates of 10 samples versus 163 variables representing the UV absorbance in the region of 200–400 nm. Each of them represents the UV spectrum for one of the eight cultivars of *Vicia faba* species, as well as the two other *Vicia* species, *Vicia sativa* and *Vicia monantha*. To assess the variation between the UV spectra of the ten different samples of *Vicia* seeds, principal component analysis (PCA) was applied using the full cross-validation method after mean centering of the UV data. PCA is an unsupervised technique for data reduction that creates a visual scatter plot known as a score plot. This plot allows for a qualitative assessment and visualization of the grouping, patterns, similarities, and variability among the samples. The resultant PCA score plot (Fig. 1a) was successful in clearly segregating the 8 samples of fava bean seeds from the two samples of *Vicia sativa* and *Vicia monantha*. The first two principal components, PC1 and PC2, explained 99% of the total variation of the data. From the scatter score plot, it was found that the samples of the eight different varieties of fava beans were separated and positioned at the left (negative) side along PC1, while *Vicia sativa* and *Vicia monantha* samples were located at the right (positive) side along PC1. These results suggest that *Vicia sativa* and *Vicia monanta* exhibit a greater degree of similarity in their UV spectra compared to *Vicia faba*. In addition, the sample of *Vicia sativa* was separated from the sample of *Vicia monantha* along the PC2, which explains only 4% of the total variation in data. This finding also confirms the high degree of resemblance between the UV spectra of *Vicia sativa* and *Vicia monantha*. Furthermore, there were 3 clusters within the *Vicia faba* samples along PC1 and PC2. The first cluster represents the Spanish cultivar LUZ sample, the second cluster represents the two new Egyptian cultivars Maryout2 (MRA) and Maryout 3 (MRB), and the third cluster contains the samples of the five traditional Egyptian cultivars: Sakha 1 and 4 (SKHA and SKHB), Giza 716 and 843 (GZA and GZB), and Masr (MSR). This interesting finding suggests the potential application of UV spectroscopy not only in the discriminations of fava bean samples from other *Vicia* species (VS and VM) but also in the discrimination between at least some of the varieties within the same species of *Vicia faba.*

**Fig. 2:**
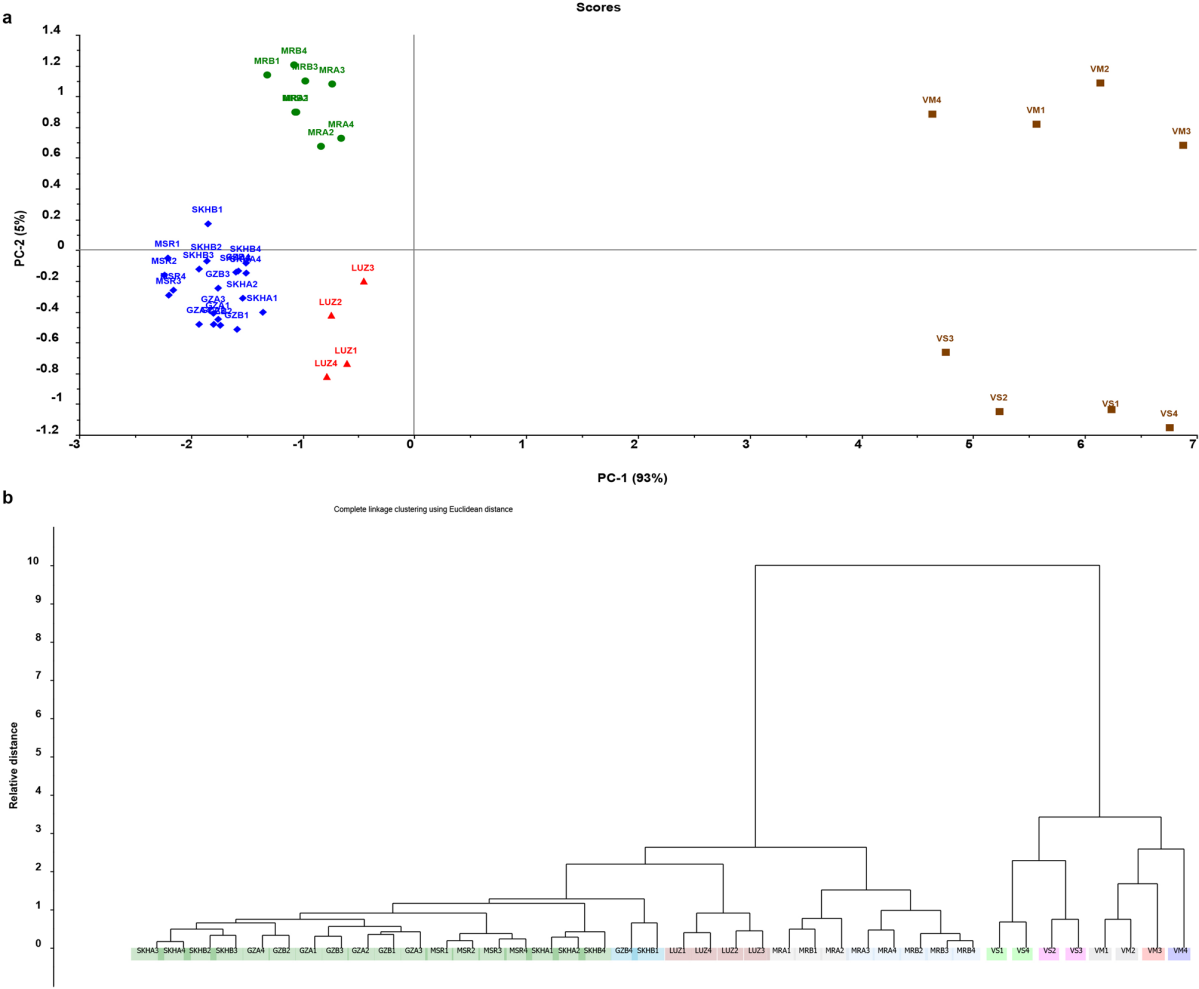
PCA score plot **(a)** and HCA dendrogram **(b)** of UV spectra of 40 samples of *Vicia sativa*, *Vicia monantha* (*n* = 8**)**, and eight varieties of *Vicia faba* (*n* = 32).

Regarding vibrational FT-IR spectroscopy, Fig. 2S presents the FT-IR spectra of ten different samples of *Vicia* seeds in the mid-IR region (4000–400 cm^-1^). While all spectra display similar overall spectroscopic profiles, there is significant variability in spectral amplitudes across samples, which was largely eliminated by applying the SNV algorithm. FT-IR is a valuable technique for identifying the functional groups present in the analyzed samples. The FT-IR spectra of all samples exhibited characteristic peaks that were indicative of various functional groups. A broad peak observed at approximately 3280 cm^-1^ corresponded to OH stretching, while absorptions at ~ 2927 and 2850 cm^-1^ were attributed to the asymmetric and symmetric stretching vibrations of methylene (-CH2) groups. Additional peaks were assigned to C ≡ N stretching at ~ 2225 cm^-1^, O-C = O stretch at ~ 1735 of triglycerides, C = O stretching at ~ 1640 cm^-1^ for amides or other compounds containing carbonyl groups, N-H-C = O at ~ 1540 cm^-1^ for amide II in protein, OH bending at ~ 1390 cm^-1^ for phenols, C-C stretching or C-O bonds of polysaccharides at ~ 1230 cm^-1^, C-O stretching of polysaccharides or C = C bending at ~ 1000 cm^-1^ (aromatic rings of cellulose), and -C = O bending at 850 cm^-1^. The main spectral peaks were ascribed to a variety of chemical components, such as water, proteins, polysaccharides, and lipids. These results are in accordance with previous studies^78–80^.

PCA exploratory analysis was also conducted on FT-IR spectroscopy data belonging to the ten samples of *Vicia* seeds. The FT-IR absorption spectral data of the ten *Vicia* samples in the region of 4000–400 cm^-1^ underwent preprocessing using the Standard Normal Variate (SNV) algorithm to eliminate or reduce the scatter effects including the baseline shift and multiplicative effects arising from particle size and packing differences, followed by mean centering prior to PCA application. PCA score plot for the FT-IR spectra was presented in Fig. 1b. The first two principal components (PC1 and PC2) accounted for 69% and 8% of the total variation in the FT-IR spectroscopy data, respectively. Similar to the findings with UV spectra, FT-IR spectra effectively discriminated between the *Vicia faba* samples and the other *Vicia* species. The *Vicia faba* samples clustered on the left (negative) side of the PC1 axis, while the two samples of *Vicia sativa* and *Vicia monantha* were positioned on the right (positive) side, indicating clear separation based on PC1. The two samples of *Vicia sativa* and *Vicia monantha* were further separated along PC2, even though PC2 accounted for only 8% of the total variance. This finding supports the notion of greater similarity between the IR spectra and chemical components of VM and VS, as previously observed with UV spectra. However, the FT-IR spectra demonstrated limitations in discrimination between the samples of varieties within the *Vicia faba* species, compared to the capabilities of UV spectra. The various fava bean varieties were clustered into only two clusters on the left half of the score plot (compared to 3 clusters in the case of UV spectra): one cluster represents all of the traditional and new Egyptian fava bean varieties, and the other cluster represents a sample of the Spanish variation Luz de Otoño. While no prior research has employed UV spectroscopy, a limited number of studies have utilized FT-IR and NIR spectroscopy to qualitatively discriminate between fava bean cultivars or the growing location/season of the fava bean samples. Johnson and coworkers employed FTIR to rapidly profile phytochemical variations between ten cultivars of Australian fava beans. They constructed a Partial least squares discriminant analysis (PLS-DA) model that was only capable of classifying the fava bean samples based on the growing year with accuracy (> 87%). Attempts to classify the fava bean samples according to the growth site using PLS-DA were less successful (59% accuracy)^79^. The same research group explored the potential application of FT-IR and NIR spectra for the prediction of antioxidant activity and key chemical components in Australian fava bean varieties. Firstly, None of the FT-IR models yielded satisfactory results for any investigated parameter. Secondly, NIR models could not predict most of the analytes except protein content, alongside rapid approximation or prediction of samples with high versus low phenolics and antioxidant capacity^80^. Combination between FT-IR absorption bands for proteins and polysaccharide, in conjunction with the mineral contents measured by ICP-MS (inductively coupled plasma mass spectrometry) was successful to discriminate white varieties from green varieties of Chinese Fava beans^52,81^.On the other hand, the principal component analysis (PCA) application to the FT-IR bands of only the protein or carbohydrate regions partially discriminated between Western Canadian fava bean varieties to some extent, while cluster analysis showing partial separation between “low tannin and regular tannin-containing” varieties^52,82^. Using NIR spectroscopy was more promising in identifying fava bean cultivars grown in various locations across China, based on spectral characteristics pertaining to protein, starches, oil and polyphenols^83^. Regarding the discrimination between different *Vicia* species, Fayek and colleagues, in a remarkable study, have used an untargeted metabolomics approach based on UPLC-MS metabolite profiling to discriminate between 16 *Vicia* species, including *Vicia faba* and *Vicia sativa*. Their findings align with our study’s results, demonstrating the effectiveness of PCA score plots based on UPLC-MS metabolite profiling data in discriminating *Vicia faba* from the other 15 *Vicia* species, including *Vicia sativa* and others^4^. Our study showed a remarkable similarity between *Vicia sativa* and *Vicia monantha* in both UV and FT-IR spectra and a clear separation from the *Vicia faba* samples spectra. In the aforementioned study, most of the studied species, including *Vicia sativa* and other species (12 out of 16 species), clustered together and failed to separate in the PCA score plot, indicating a similar metabolome between most *Vicia* species, and only *Vicia faba* and three other species were successfully separated and have shown distinctive metabolite profiles from the other 12 *Vicia* species (*Vicia sativa* and others)^4^. These findings suggest that UV and FT-IR spectroscopy could serve as viable alternatives to UPLC-MS for discriminating *Vicia faba* from other *Vicia* species, offering advantages such as lower costs, easier preparation and operation, and simpler data acquisition and analysis compared to UPLC-MS data.

Based upon our preliminary exploratory data analysis of both UV spectra and FT-IR spectra, it seems that UV spectroscopy exhibited superior discriminatory capabilities compared to FT-IR spectra. Consequently, we opted to proceed with UV spectra for the development of more detailed unsupervised discrimination and clustering models, as well as supervised SIMCA and PLS-DA classification models, particularly for discriminating among some of the more closely related varieties within the same species, *Vicia faba*.

### Building unsupervised PCA and HCA models based on the UV-spectroscopy of *vicia* seeds

**Fig. 3:**
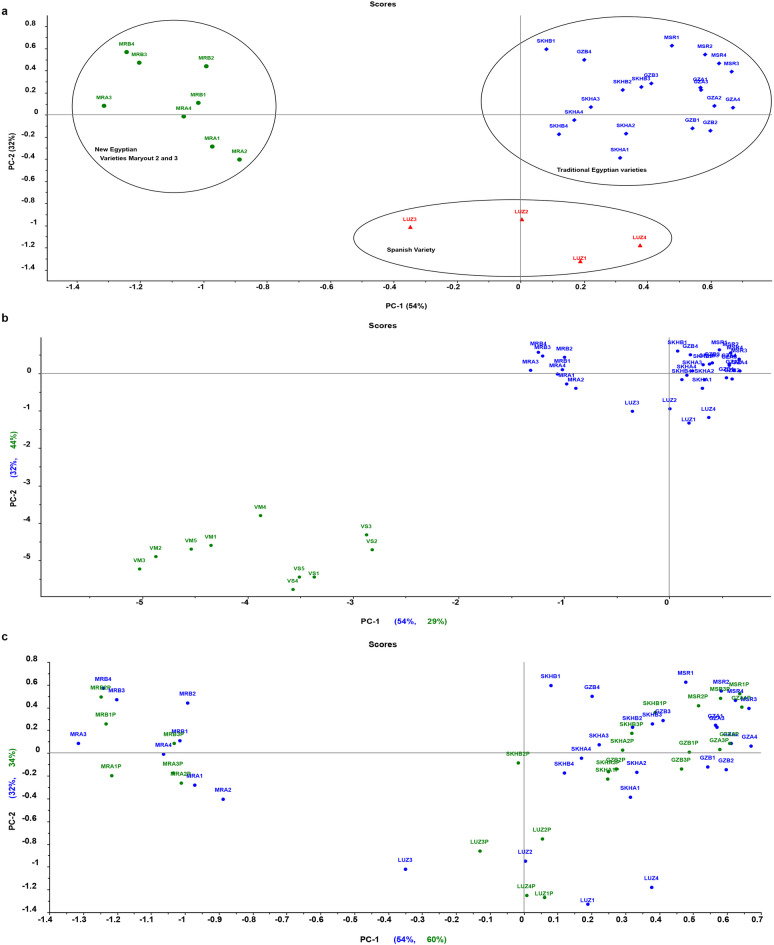
The PCA model of the eight fava bean varieties based on the UV spectra of the training set (*n* = 32 samples) **(a)**. This model was challenged by 10 non-fava bean samples from *Vicia sativa* (VS) and *Vicia monantha* (VM) and all of them clustered away from the training set samples (blue dots, *n* = 32) and appear as outliers (green dots, *n* = 10) in **(b).** This model was also challenged by a validation set (*n* = 25, the green dot) representing the eight fava bean varieties and all of them clustered correctly with their respective cluster **(c)**.

The methanolic extracts of forty *Vicia* seed samples, comprising four samples each of *Vicia sativa* and *Vicia monantha*, and thirty-two samples distributed across eight fava bean varieties (four samples per variety), were analyzed for their UV absorbance spectra in the 200–400 nm range **(**Fig. 3S**).** The resulting data were subjected to unsupervised clustering techniques, including principal component analysis (PCA) and hierarchical cluster analysis (HCA), following mean centering. The PCA score plot **(**Fig. 2a) revealed a clustering pattern, similar to the results of previous preliminary exploratory analysis, displaying five well-separated clusters. Three of these clusters were closely grouped on the left (negative) side of the plot, representing the five commercial traditional Egyptian fava bean varieties, the Spanish variety Luz de Otoño, and the new Egyptian varieties Maryout 2 and 3, respectively. The remaining two clusters were located on the right (positive) side of the plot and corresponded to *Vicia sativa* and *Vicia monantha*.

Moreover, hierarchical cluster analysis (HCA) was applied to classify the samples based on the similarities and differences among their UV spectral data. The resulting HCA dendrogram **(**Fig. 2b**)** revealed a clear division of all *Vicia* seed samples into two main clusters. The first cluster was further divided into two subclusters, each corresponding to *Vicia sativa* and *Vicia monantha*, respectively. The second main cluster was also subdivided into three subclusters: one for the five traditional Egyptian fava bean varieties, another for the two new Egyptian varieties Maryout 2 and 3, and a third for the Spanish variety Luz de Otoño. The clustering pattern observed in HCA corroborated the findings of PCA, supporting two key conclusions. Firstly, a greater similarity was evident between *Vicia sativa* and *Vicia monantha*, in contrast to their clear dissimilarity with *Vicia faba*. Secondly, on one hand, a notable difference was observed between the traditional commercial Egyptian fava bean varieties and the Spanish variety Luz de Otoño. On the other hand, both the traditional Egyptian fava bean varieties and the Spanish variety were clearly distinguishable from the two new Egyptian varieties, Maryout 2 and 3.

To further assess the effectiveness of UV spectra in conjunction with multivariate statistical models for identifying and classifying the three previously defined classes of Fava bean samples, as well as discriminating them from non-fava bean samples like *Vicia sativa* and *Vicia monantha*, a series of models were developed. A training set consisting of the previously employed UV spectra of 32 samples (four samples per each variety), including 4 samples for the Spanish variety Luz de Otoño, 8 samples for the two new Egyptian varieties Maryout 2 and 3, and 20 samples for the five traditional commercial Egyptian fava bean varieties, was subjected to principal component analysis (PCA) to construct a specific PCA model for the varieties of *Vicia faba* species. As anticipated, the PCA model trained exclusively on fava bean samples **(**Fig. 3a**)** effectively separated these samples into three distinct clusters: one cluster for the five traditional Egyptian fava bean varieties, another cluster for the two new Egyptian varieties Maryout 2 and 3, and a third cluster for the Spanish variety Luz de Otoño. Subsequently, this PCA model was challenged with 10 non-fava bean samples from *Vicia sativa* and *Vicia monantha*, which were all identified as outliers on the PCA score plot **(**Fig. 3b**)**. Furthermore, the trained PCA model was challenged with a validation set comprising 25 samples representing the eight fava bean varieties **(**Fig. 3c**).** Each of the 25 test samples was accurately clustered with its corresponding cluster within the training set samples, apparently demonstrating the model’s robust discrimination power. Our findings revealed that the UV spectra of the five traditional Egyptian varieties samples are more similar to one another than to the new Egyptian varieties Maryout 2 and 3, as well as the Spanish variety Luz de Otoño. In this regard, previous comparative metabolite profiling based on LC-MS analysis conducted by Mekky and coworkers on the seeds and sprouts of three traditional Egyptian fava bean varieties, including Giza 834, Sakha 3, and Nubaria 3, revealed a remarkable similarity in their qualitative chemical profiles^8^. On the other hand, fava beans have been shown to exhibit significant genetic variation in terms of seed composition, size, and floral biology^2,13,30^. The composition of major polyphenol groups was investigated in ten varieties of immature fava bean seeds cultivated in Chile, including Luz de Otoño and nine others. Their study identified significant differences among these varieties, highlighting the ample phenotypic variability available for future selection studies focused on traits such as nutritional value, taste, and ease of production. Moreover, the later study also revealed an interesting finding about Luz de Otoño, the Spanish variety, which exhibited the lowest concentration of total phenolics and the highest levels of condensed tannins among all the studied varieties^2^. Regarding the new Egyptian Maryout varieties, few previous studies comparing them (in some traits) to commercial traditional Egyptian varieties have revealed differences in certain traits like morphological characteristics, mean seed yield, and protein content^84,85^. Further chemical investigations are warranted to comprehensively elucidate the distinctions between these varieties.

### Building supervised SIMCA and PLS-DA predictive models for classification of fava bean seeds

**Fig. 4:**
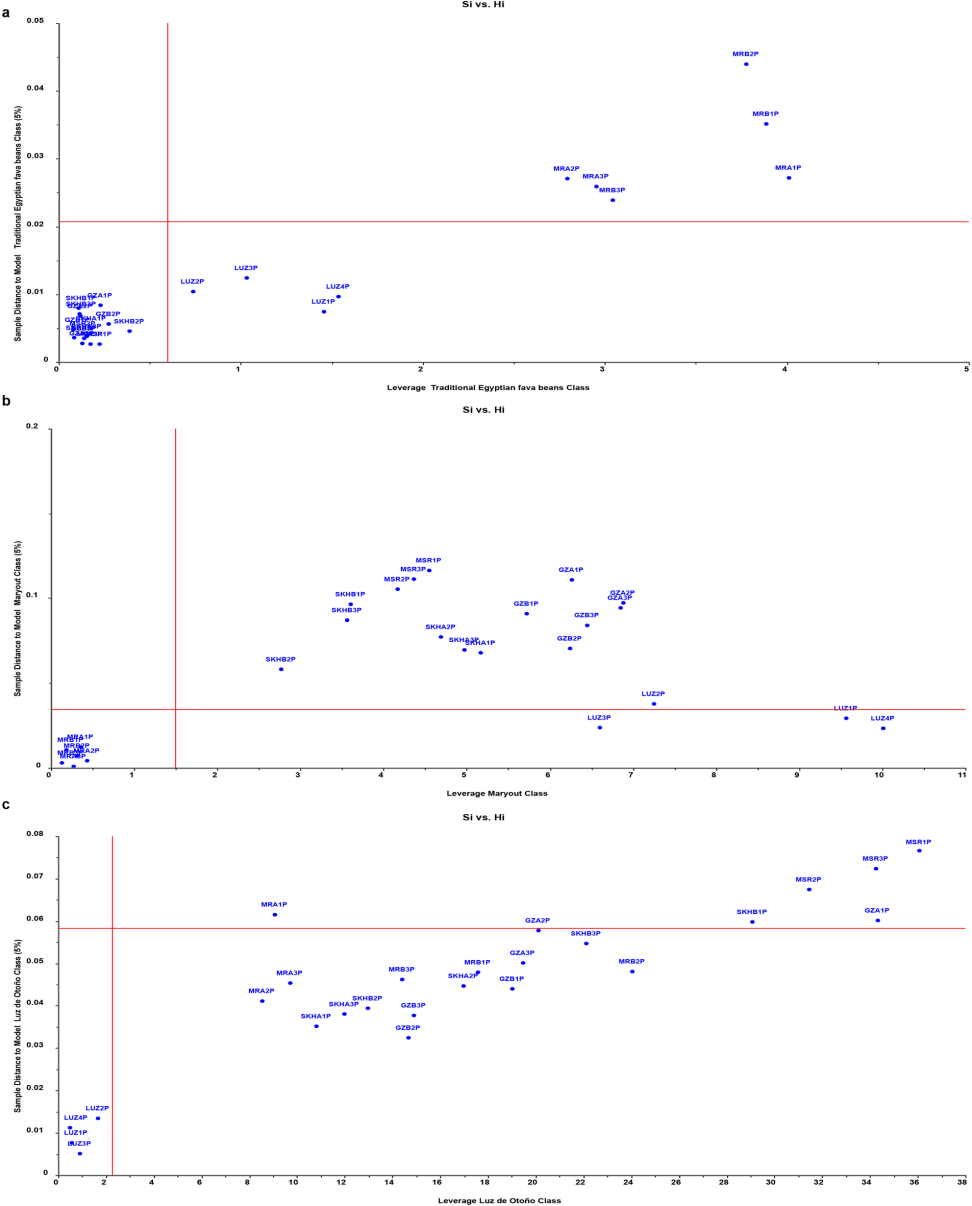
Three Si vs. Hi plots for the validation samples (from the eight fava bean varieties, *n* = 25) representing the closeness and classification of these samples to one of the three models’ classes of fava bean varieties. The samples that belong to a certain model class lie in the left lower quadrant with a small distance and leverage to the model for which they belong. The validation samples from the five traditional Egyptian cultivars lie in the lower left quadrant of the traditional fava bean class model, while Maryout and LUZ samples lie outside this quadrant with high leverage and/or distance to the tradition Egyptian fava bean model **(a).** Only Maryout validation samples lie in the lower left quadrant of Maryout class (new Egyptian varieties), while all other varieties lie outside this quadrant **(b)**. Finally, only LUZ validation samples lie inside the lower left quadrant of the Spanish variety Luz de Otoño class whereas all Egyptian verities lie outside this quadrant **(c).**

**Fig. 5:**
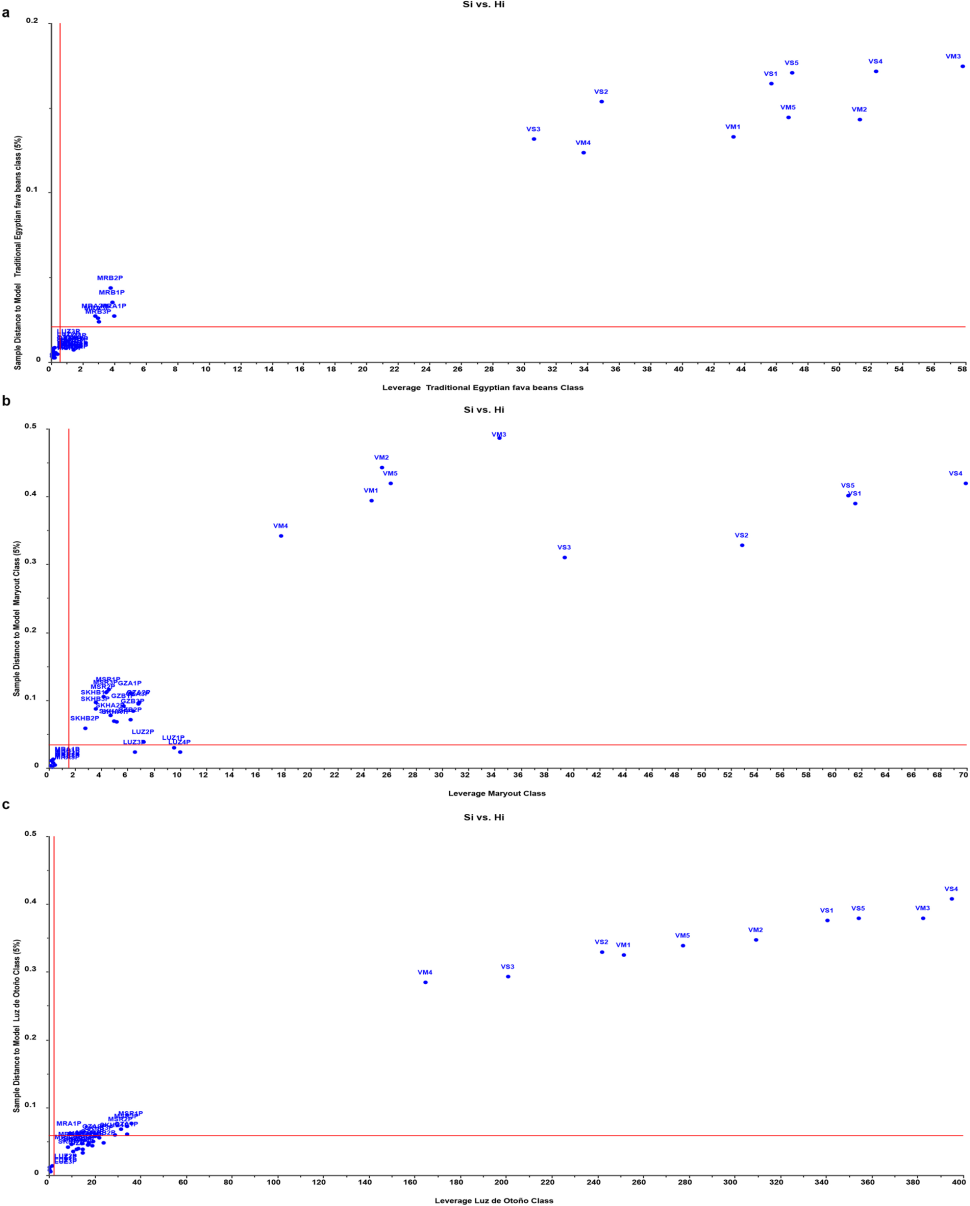
Three Si vs. Hi plots shows that all non-fava bean samples (*n* = 10) of *Vicia sativa* and *Vicia monantha* have both high model distance and leverage. So, they do not belong to any of the three fava bean classes and appeared as very outliers at the upper right quadrant of the Si vs. Hi plots of traditional Egyptian fava bean class model **(a**), Maryout class model **(b)**, and the Spanish variety Luz de Otoño class model **(c).** The other samples in the figure represent the validation set of fava bean varieties (*n* = 25).

To further investigate the previous results concluded by the unsupervised model of PCA, the supervised pattern recognition methods SIMCA and PLS-DA were employed to build predictive classification models. The Soft Independent Model of Class Analogy (SIMCA) technique is a pivotal chemometric tool capable of categorizing samples into pre-established groups, assigning new objects to the class exhibiting the greatest similarity. SIMCA is strongly based on PCA because each class is defined by an individual PCA. The SIMCA classification process comprises two distinct phases: the training stage, wherein individual models of the classes are constructed, and the testing or validation stage, during which new samples (not used in the training phase) are categorized within the established class models to assess the model’s efficiency. In our study, during the training phase, 3 distinct classes were established using independent PCA models for each single class. These classes represented the five traditional Egyptian fava bean varieties (20 samples, 4 samples per variety), the Spanish variety Luz de Otoño (4 samples), and the two new Egyptian Maryout 2 and 3 varieties (8 samples, 4 samples per variety), respectively. Subsequently, a validation set composed of 25 samples representing each of the eight fava bean varieties and 10 samples from non-fava bean species (*Vicia sativa* and *Vicia monantha*) were used. As shown in the confusion matrix for SIMCA classification in the upper half of Table 2, the model achieved 100% classification accuracy for all three classes, with no misclassifications observed between the Egyptian five traditional fava bean varieties class, the Spanish variety Luz de Otoño class, or the two new Egyptian Maryout 2 and 3 varieties class. Crucially, the model correctly rejected all 10 non-target *Vicia sativa* and *Vicia monantha* samples during validation phase, demonstrating excellent specificity. Further details on the classification of validation samples (not used in model training) are reported in the SIMCA classification table (Table 1S). It shows that the 25 validation samples representing different fava bean varieties were correctly classified as members of their corresponding classes. Conversely, all the 10 non-fava bean samples from *Vicia sativa* and *Vicia monantha* were not assigned to any of the 3 fava bean classes. Each sample is assigned to a certain class based on metric distances unique to each class, such as Si and Hi, which estimate sample-to-model distance and sample farness from the model center (leverage). Three **Si vs. Hi** plots in Fig. 4a, b, **and c** were used to evaluate the classification results, where in case a sample belonged to a certain class, it should fall within the class membership limit, on the left below the horizontal line. The validation samples representing traditional commercial Egyptian fava bean varieties, as well as the new Egyptian varieties Maryout 2 and 3, and the Spanish variety Luz de Otoño, were all found to lie within the membership boundaries with small distance and leverage from their respective models, demonstrating the high sensitivity and predictability of the model. Moreover, the SIMCA model showed good specificity, as all non-fava bean samples of *Vicia sativa* and *Vicia monantha* were not classified into any of the three classes and appeared as very outliers at the upper right quadrant of the Si vs. Hi plots **(**Fig. 5a, b, **and c).** Additionally, the model distance between each pair of models was estimated to clarify the model’s discriminative potential to discriminate the spectral signals of the 3 classes. This provides a measure of how separable the class models are. Good class separation is indicated by a distance greater than three, implying a high likelihood of distinguishing the classes from one another. In this study, it is noteworthy that the class models exhibited considerable differences, resulting in interclass distances of approximately 89 and 32 for the two Maryout varieties class and the Spanish variety class, respectively, when compared to the class of traditional fava bean varieties **(see details of model distance in** Fig. 4S**)**. Furthermore, the discrimination power for all variables was greater than 2 (most of them had more than 3) between any pair of classes, reflecting the discriminatory capability of the constructed SIMCA model in distinguishing among the three classes of fava beans **(See details of discrimination power in** Fig. 5S**)**. The ability of the SIMCA model to classify and discriminate between the UV spectra of the 3 classes of fava beans and consider all non-fava bean samples as extreme outliers corroborates and validates the previously constructed unsupervised PCA and HCA models. While the model performed well on this dataset, further validation with larger sample sets, including geographically diverse origins, would strengthen generalizability.

**Fig. 6:**
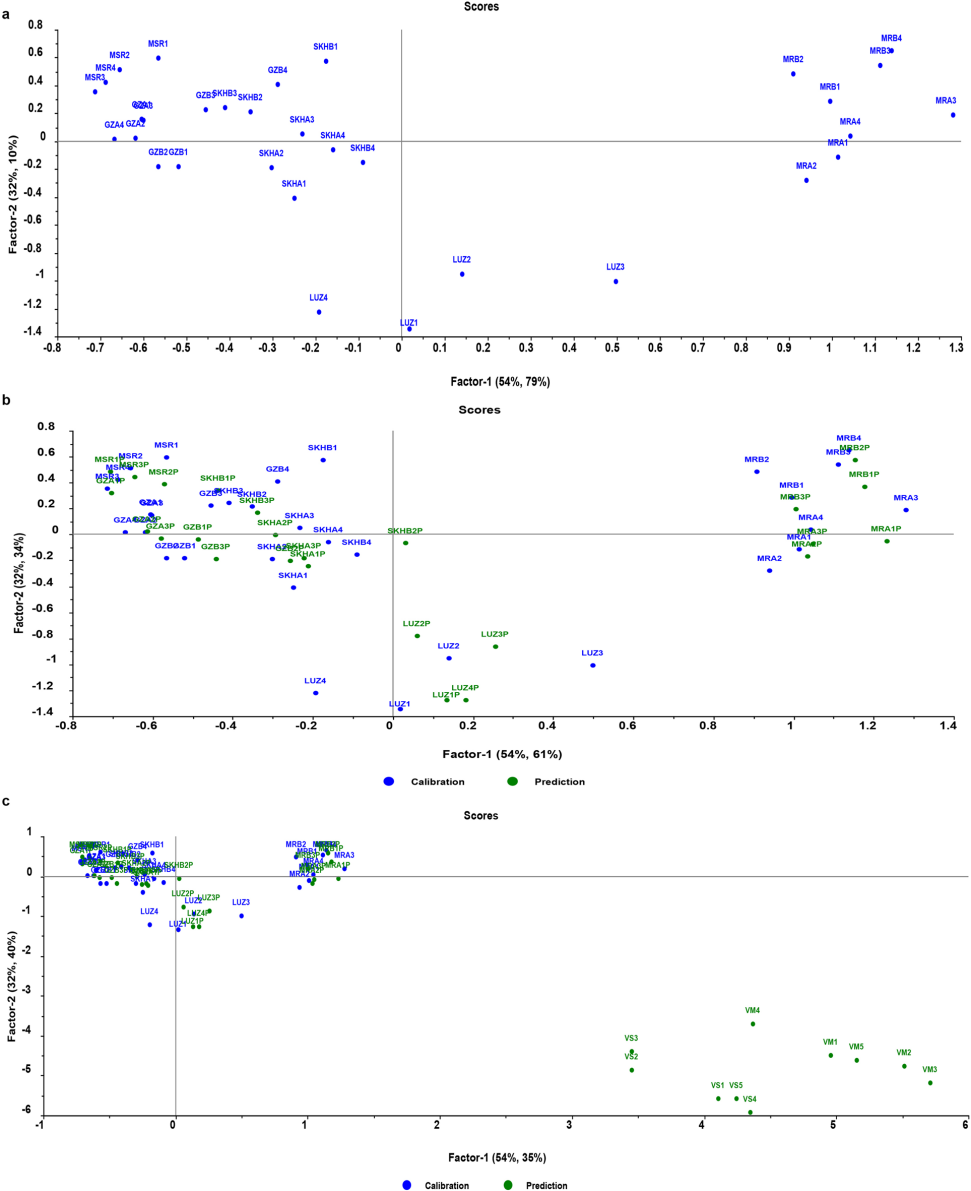
The Score plot of PLS-DA model shows how samples are projected onto the first 2 factors and how classes are well separated. The calibration set (blue dots) (*n* = 32) of fava beans formed three distinct clusters in the score plot, signifying well separated 3 classes along factors 1 and 2 **(a)**. Panel **(b)** (*n* = 57) represents the validation unknown samples (green dots, *n* = 25), which were accurately classified and clustered closely with the training samples (blue dots, *n* = 32) of their correct class. On the other hand, **panel (c)** (*n* = 67) shows that the non-target species samples (green dots VS and VM at the lower right quadrant, *n* = 10) were clustered away far from the three classes of fava bean varieties.

The supervised discriminant method, partial least squares discriminant analysis (PLS-DA), was implemented to augment the separation between the three classes of fava beans, namely: the five traditional Egyptian fava bean varieties, the two new Egyptian varieties Maryout 2 and 3, and the Spanish variety Luz de Otoño. A PLS-DA calibration model with seven latent variables was created using the training set of the eight fava bean varieties spectral data that were previously used and exploiting the leave-one-out-cross validation (full cross validation method). The score plot representing the first and second latent variables for the calibration set, as depicted in Fig. 6a, demonstrates the attainment of good class separation, characterized by the formation of three distinct clusters along both factors 1 and 2. The samples of the two new Egyptian fava bean varieties appeared at the far right side of the score plot, while the traditional five varieties were located at the left side of the plot, and the Spanish variety Luz de Otoño appeared at the lower middle part of the score plot. Furthermore, Fig. 6b shows the accurate classification and clustering of the validation set samples of fava bean varieties to their correct classes of fava bean. While Fig. 6c shows that the non-fava bean samples from other *Vicia* species were clustered away from the three classes of fava bean varieties. Similar to what observed with the SIMCA classification results, the lower half of Table 2 presents the PLS-DA confusion matrix, which highlights the PLS-DA model’s strong discriminatory performance among the three classes of fava bean varieties. Figure 6S showed the predicted with deviation plot for all the non-fava bean samples as well as the validation set samples of fava bean varieties. All samples from fava bean varieties within the validation set were accurately assigned to their respective classes, achieving 100% classification accuracy. On the other hand, all 10 samples from *Vicia sativa* and *Vicia monantha* were predicted as outliers with very high deviation, demonstrating the high specificity of the PLS-DA model.

While the results of our classification models are very promising on the evaluated dataset, their generalizability may be constrained by the relatively small sample size and limited diversity, particularly in geographic origin (e.g., only one Spanish variety was represented). Future studies should extend our model by training and validating it using larger and more varied sample sets, including broader representation across cultivars from diverse geographical origins, to ensure robust applicability. However, our sample size per variety/species aligns with previous chemometric studies that utilized spectroscopy and mass spectrometry data to develop initial methods and models for discrimination among *Vicia* species and varieties. For instance, in a remarkable study, Fayek and colleagues applied multivariate techniques to LC-MS data for classifying 16 *Vicia* species from over four European countries using only 3 samples per species^4^. Similarly, other studies applying IR included 3 samples per variety for 6 fava bean varieties grown in one place in Western Canada^82^and 10 samples per variety for 10 fava bean varieties grown in two locations in south Australia^80^. Despite the limited sample size, the models developed in this study achieved excellent classification performance (100% accuracy) when grouping the 8 varieties into three key classes: class1: the five traditional Egyptian fava bean varieties (20 samples training/15 samples validation); class2: the Spanish variety Luz de otoño (4 samples training/4 samples validation); and Class 3: the two new Egyptian varieties of Maryout 2 and 3 (8 samples training/6 samples validation). Crucially, the accurate classification of the Spanish variety provides a proof of concept for the transferability of the model to other global varieties outside Egypt. Moreover, the models excluded all 10 samples of non-fava bean *Vicia* species with perfect specificity, demonstrating robust discriminative power despite the sample size. Finally, we think that the limited inclusion of non-Egyptian fava bean varieties (only one Spanish variety) in this study, along with the fact that previous research has also largely focused on local varieties with small samples sizes, underscores the need for future multinational collaborative research. Such efforts could evaluate the geographical robustness of discrimination models and enhance their generalizability. Nevertheless, our discrimination and classification models provide significant value in discriminating and classifying some of the very popular competing varieties in the Egyptian market, particularly the traditional Egyptian varieties that are widely used and newer, competing varieties in the Egyptian market, such Maryout 2 and 3, and the Spanish variety Luz de Otoño. These varieties mainly compete in terms of seed and crop yield, nutritional quality (protein content), maturing time, and disease resistance under different environmental conditions^10,84–89^. Furthermore, the classification models developed in this study for *Vicia* varieties offer several potential applications, such as seed authentication and prevention of adulteration. Certain varieties may be misrepresented, intentionally or unintentionally, during trade or storage. Ensuring the correct identification and exchange of fava bean varieties by farmers and traders is crucial to avoid economic losses, compromised crop performance, and quality issues. Another important application of the classification models of fava bean varieties lies in optimizing agronomic decision-making and plant breeding programs, since some varieties may be more suitable for specific climates, soils, or agronomic practices (e.g., drought resistance, disease tolerance). For example, the new Egyptian varieties of Maryout have been shown to outperform the traditional Egyptian varieties in seed yield and protein content, particularly under drought and rainfed conditions^84,85^. Another example is the reported yield of the early-maturing Spanish variety Luz de Otoño which is lower than the traditional and new Egyptian varieties^84–88^. Our study has shown that the new Egyptian varieties of Maryout can be easily discriminated from the traditional Egyptian varieties and the Spanish variety based on rapid, low cost, and simple models of spectroscopic (UV) analysis. A further application of these models is in the food industry and nutrition field, as fava beans and *Vicia* seeds are considered excellent sources of vegetarian protein and the nutritional value such as protein content varies significantly across varieties. Low-cost and rapid classification of varieties may support the selection of superior variety for food products and nutritional planning. It is also worth mentioning the importance of classification models for *Vicia* seeds in the preservation of endemic species and the protection of traditional cultivars, which may be at risk of being lost or mixed with commercial lines, especially due to competition from newer and imported varieties in the Egyptian market.

**Table 2:** Confusion matrix table of SIMCA and PLS-DA models based on the training and validation data sets. The total samples of training and validation sets is 67 samples. Class (1): the five traditional Egyptian fava bean varieties including (Sakha 1, Sakha 4, Giza 716, Giza 843, and Masr 1). Class (2): the Spanish variety Luz de otoño. Class (3): the two new Egyptian varieties Maryout 2 and 3. Non-target species: *Vicia sativa* and *Vicia monantha*.

Confusion matrix for SIMCA classification											
Training (*n* = 32)						Validation (*n* = 35)					
	Class (1)	Class (2)	Class (3)	Rejected	Total		Class (1)	Class (2)	Class (3)	Rejected	Total
Class (1)	20	0	0	-	20	Class (1)	15	0	0	0	15
Class (2)	0	4	0	-	4	Class (2)	0	4	0	0	4
Class (3)	0	0	8	-	8	Class (3)	0	0	6	0	6
Non-target species	-	-	-	-	0	Non-target species	0	0	0	10	10
Total	20	4	8	0	32	Total	15	4	6	10	35

**Confusion matrix for PLS-DA classification**											
**Training (n = 32)**						**Validation (n = 35)**					
	Class (1)	Class (2)	Class (3)	Rejected	Total		Class (1)	Class (2)	Class (3)	Rejected	Total
Class (1)	20	0	0	-	20	Class (1)	15	0	0	0	15
Class (2)	0	4	0	-	4	Class (2)	0	4	0	0	4
Class (3)	0	0	8	-	8	Class (3)	0	0	6	0	6
Non-target species	-	-	-	-	0	Non-target species	0	0	0	10	10
Total	20	4	8	0	32	Total	15	4	6	10	35

### Total phenolics, flavonoids and DPPH radical scavenging activity

The total phenolics, flavonoids, and DPPH radical scavenging activity of the 8 varieties of fava beans, as well as the 2 other *Vicia* species, were comprehensively summarized in Table 3. The total phenolic content of the analyzed fava bean varieties ranged from 1.88 mg GAE/g extract for the Luz de Otoño variety to 39.88 mg GAE/g extract for the Sakha 4 variety, with an average of 22.07 mg GAE/g extract. Concurrently, the total flavonoid content exhibited variation ranging from 0.57 mg QE/g extract for Luz de Otoño to 11.56 mg QE/g extract for Giza 843 variety, with an average of 7.52 mg QE/g extract. On the other hand, the *Vicia sativa* and *Vicia monantha* species demonstrated higher levels of total phenolics and flavonoids compared to all fava bean varieties. These results are consistent with previous studies where a couple of reports by Amarowicz and colleagues have determined the total phenolics in Polish cultivars to be 23.9 and 56 mg GAE/g, respectively^21,64^. Furthermore, the total phenolics of three traditional Egyptian cultivars of fava beans, including Nubaria3, Giza843, and Sakha3, were estimated to be in the range of 21.8 mg GAE/g for Nubaria to 42.36 mg GAE/g for Sakha3 ^8^. Moreover, a couple of studies have determined the range of total phenolics in a large number of Tunisian genotypes of fava bean seeds to be 16.98 to 67.47 mg GAE/g and 10.9 to 19.86 mg GAE/g, respectively^12,13^. It is also worth mentioning that the Spanish variety Luz de Otoño scored the lowest levels of both phenolics and flavonoids in comparison to the Egyptian varieties in our study. A previous study corroborates this observation, reporting a total phenolic content of 0.82 mg in the fresh immature seeds of Luz de Otoño. Moreover, the Luz de Otoño variety exhibited the lowest total phenolic content among the ten fava bean varieties from Chile, Syria, and Spain examined in the same study^2^. In addition, a previous study reported the total phenolic content of *Vicia sativa* and *Vicia monantha* to be 67.35 and 76.37 mg/g, respectively^90^. Regarding flavonoids in previous studies of fava beans, it was estimated in a couple of studies on a large number of Tunisian genotypes to be in the range of 5.25 to 6.96 mg QE/g and 5.19 to 9.3 mg RE/g, respectively^12,13^. On the other hand, it was reported that the total flavonoid content of *Vicia sativa* and *Vicia monantha* was much greater, at 47.34 and 65.23 mg/g, respectively^90^. The prevalence of phenolic compounds in plant species, particularly in legumes, is well-established and contributes substantially to their antioxidant capacity^91^. Our findings corroborate the pivotal role of these phenolic compounds in augmenting the health-promoting properties of *Vicia* seeds. The observed variations in the levels of phenolics and flavonoids among various fava bean varieties have been documented in previous studies, highlighting the intricate interplay between genetic and environmental factors in determining the production of these compounds in different fava bean genotypes^12,13,92^.

The antioxidant capacity of *Vicia faba* seeds, as measured by DPPH radical scavenging activity, exhibited a low antioxidant capacity (percentage radical scavenging activity %RSA) ranging from 2.81% for Luz de Otoño to 25.05% for G843 at a concentration of 100 µg/ml. However, the %RSA notably enhanced with increasing the concentration of extract to be in the range of 40.91% for Luz de Otoño to 88.79% for Giza 843 variety at 2 mg/ml. Among the fava bean varieties, Giza 843 demonstrated the most potent antioxidant activity with an IC50 value of 316.02 µg/ml, whereas Luz de Otoño exhibited the least antioxidant capacity with an IC50 value of 3232.52 µg/ml. Our results comply with previous reports^8,21,64^ which determined the antioxidant capacity of different fava bean cultivars. Mekky and colleagues determined the percentage radical scavenging activity of three different Egyptian fava bean cultivars at 100 µg/ml to be less than 25% with the highest value assigned to Giza 843 followed by Sakha3 and finally, Nubaria3. The antioxidant capacity of the methanolic extract of fava bean seed coat was reported to be higher than our results, with values of 44.28% and 61.05% at concentrations of 100 and 200 µg/ml, respectively^93^. Moreover, according to a previous study, fava bean pods showed superior antioxidant capacity compared to our results, with IC50 of 87.35 µg/ml and DPPH scavenging percentage of 65.7 at 250 µg/ml^56^. It is worth mentioning that the total phenolics in these studies were much higher than ours, which can account for the differences in antioxidant capacity. Furthermore, the variation of phenolic composition is not only dependent on genotype and environmental factors but is also influenced by the maturity stage and the used part of the plant (i.e., pods vs. seeds**)**^13^. In addition, the antioxidant capacity of both *Vicia sativa* and *Vicia monantha* was higher than any fava bean variety which can be attributed to the high phenolic content of these two species. In previous literature, a powerful antioxidant capacity has been reported for the ethanol extract of *Vicia sativa*^23^. Moreover, the polyphenol extract of *Vicia sativa* was superior to soybean and butylated hydroxytoluene in scavenging DPPH radicals^26^. The obtained results could be attributed to the presence of natural antioxidant phytochemicals like phenolics and flavonoids in *Vicia* seeds. These phytochemicals possess multiple hydroxyl groups in their molecular structure that can reduce or neutralize DPPH radicals through multiple mechanisms. This free radical scavenging activity might be valuable not only for promoting health and preventing disease but also in preserving foodstuffs, pharmaceutical products, and cosmetics^94^.

**Table 3:** Total phenolic content, total flavonoid, and DPPH radical scavenging activity of *Vicia sativa*, *Vicia monantha*, and eight varieties of fava beans.

plants	Total polyphenols mg/g extract	Total Flavonoids mg/g extract	DPPH %RSA at 100 µg/ml	DPPH %RSA at 2000 µg/ml	IC50 µg/ml
*Vicia sativa*	61.63 ± 3.52	42.35 ± 2.42	27.40 ± 0. 93	92.29 ± 0.70	267.41 ± 8.29
*Vicia monantha*	69.67 ± 2.79	44.49 ± 3.06	31.10 ± 1.60	94.80 ± 1.36	225.14 ± 11.69
Sakha1	19.74 ± 0.89	7.36 ± 0.42	15.41 ± 0.59	84.49 ± 2.33	369.47 ± 37.58
Sakha 4	39.88 ± 1.89	10.46 ± 0.51	23.75 ± 0.73	88.22 ± 1.67	333.74 ± 13.04
Giza 843	21.19 ± 1.13	11.65 ± 0.98	25.05 ± 0.64	88.79 ± 1.61	316.02 ± 19.70
Giza 716	18.34 ± 0.94	6.63 ± 0.55	18.86 ± 0.81	75.22 ± 0.80	571.64 ± 41.11
Masr	16.62 ± 0.50	5.53 ± 0.32	19.60 ± 0.26	76.82 ± 1.56	526.41 ± 58.50
Luz de Otoño	1.88 ± 0.12	0.57 ± 0.18	2.81 ± 0.62	40.91 ± 2.42	3232.52 ± 482.14
Maryout 2	30.59 ± 2.33	9.54 ± 0.67	22.72 ± 1.45	85.45 ± 1.18	378.97 ± 21.12
Maryout 3	28.33 ± 0.93	8.43 ± 0.23	21.38 ± 0.77	83.08 ± 0.79	437.41 ± 26.89

Phytochemical variation among *Vicia* cultivars and species, such as the variation in phenolics and flavonoids content, may influence their bioactivity and health-promoting capabilities. Interestingly, our discrimination models are beneficial in discriminating certain species, such as *Vicia sativa* and *Vicia monantha*, which are rich in phenolics and flavonoids from other *Vicia faba* varieties. Furthermore, they offer significant value in discriminating the Spanish fava bean variety Luz de Otoño, which has a lower content of phenolics and flavonoids compared with the Egyptian varieties. Rapid discrimination and classification models based on low-cost UV spectroscopic analysis supports the field of developing phytopharmaceuticals and functional foods by helping to avoid varieties with low levels of these compounds (e.g., Luz de Otoño) and select the varieties and species that are higher in these compounds. Future studies should expand on optimization of these models for the field of developing phytopharmaceuticals and plant-based health products. The phytochemical parameters analyzed in this study are highly relevant to research in food science, nutritional analysis, functional foods and phytopharmaceuticals development. Moreover, our research may be the starting point for selecting which *Vicia* variety or species might be used in health-related problems. For example, a recent study selected the fava bean cultivar “Sakha 3” for neuroprotective and anti-inflammatory evaluation in Parkinson model^18^ based on prior estimation of phenolic content of three Egyptian cultivars in an earlier study by the same group^8^demonstrating how phytochemical estimation, such as ours, can guide variety selection for health-related applications research. Furthermore, the results of phytochemical parameters in this study might guide the food and pharmaceutical industries in the rational selection of the proper *Vicia* species or fava bean variety for development of novel functional foods or phytopharmaceuticals, with the traditional fava bean cultivars Sakha 4 and Giza 843 as well as the *Vicia sativa* and *Vicia monantha* species being the best. Finally, integration of spectroscopy with multivariate statistics for discrimination among *Vicia* seeds, and phytochemical estimation might be of interest to wide range of disciplines and support application in many fields, such as applied spectroscopy, crop authentication and prevention of adulteration, food science and nutritional analysis, agronomy optimization, plant breeding, plant-based health products and developing phytopharmaceuticals, and biodiversity.

## Conclusion

In the present study, UV and FT-IR spectroscopy were used in combination with multivariate statistical tools not only for the sake of distinguishing *Vicia faba* seeds from other *Vicia* legumes such as *Vicia sativa* and *Vicia monantha*, but also for discrimination between some varieties or cultivars of the same species of *Vicia faba*. Preliminary exploratory analysis showed that both techniques could differentiate fava beans from other *Vicia* legumes. However, when it came to the varieties of fava beans, the UV was superior to FT-IR in discrimination between the varieties. Furthermore, PCA and HCA models based on the UV spectra of fava bean varieties were capable of separating the 8 fava bean varieties into 3 informative clusters. The first cluster contained the five commercial traditional Egyptian fava bean varieties, whereas the second cluster contained the two new Egyptian varieties, Maryout 2 and 3. The third cluster contained the Spanish fava bean variety, Luz de Otoño. The supervised classification models, SIMCA and PLS-DA, further validated the results and showed well separation between the three classes. This study demonstrated for the first time that UV spectroscopy could serve as a simple, fast, and low-cost discriminatory tool for some important *Vicia* seeds. In addition, the phenolic and flavonoid phytochemical contents, as well as the DPPH radical scavenging activity were determined for different *Vicia* seeds in this study. Among the varieties of fava bean analyzed, the Spanish variety Luz de Otoño had the lowest total phenolic content, while the traditional Egyptian variety Sakha 4 had the highest level. Regarding flavonoids and DPPH radical scavenging activity, the traditional variety Giza 843 was the highest, while the Luz de Otoño variety was the lowest. On the other hand, both *Vicia sativa* and *Vicia monantha* were superior to the eight varieties of fava beans in terms of phenolics, flavonoids, and DPPH radical scavenging activity. These results might guide the rational choice of the suitable fava bean varieties and *Vicia* species for developing functional foods and phytopharmaceuticals, with the traditional fava bean cultivars Sakha 4 and Giza 843 as well as the *Vicia sativa* and *Vicia monantha* species being the best.

## Supplementary Information

Below is the link to the electronic supplementary material.

Supplementary Material 1

## Data Availability

All datasets generated or analyzed during this study are available from the corresponding author on reasonable request.
