# Supplementary material for: Using UV and FTIR spectroscopy for discrimination among vicia seeds with emphasis on UV based multivariate modelling
Source: Sci Rep. 2025 Sep 23;15:32652. doi: 10.1038/s41598-025-17113-y (PMC12457650; doi:10.1038/s41598-025-17113-y)
Supplement: Supplementary file 1 — Supplementary Material 1 [file 41598_2025_17113_MOESM1_ESM.pdf]

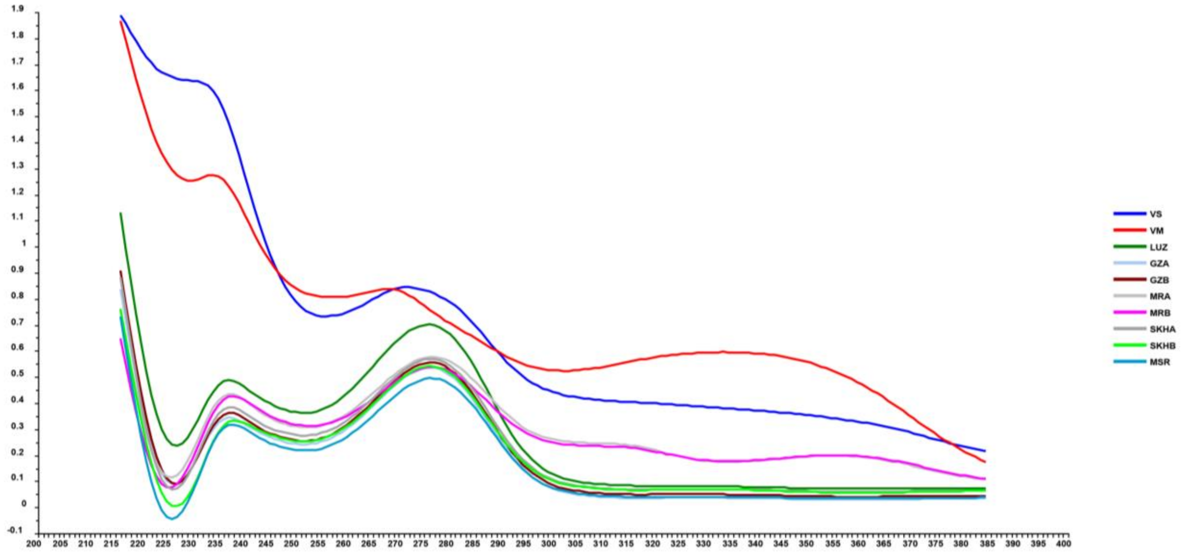

Figure 1S. The UV absorbance spectra of the methanol extracts of ten *Vicia* samples were measured in the range of 200 – 400 nm. Each of them represents the UV spectrum for one of the eight cultivars of *Vicia faba* species, as well as the two other *Vicia* species, *Vicia sativa* and *Vicia monantha*.

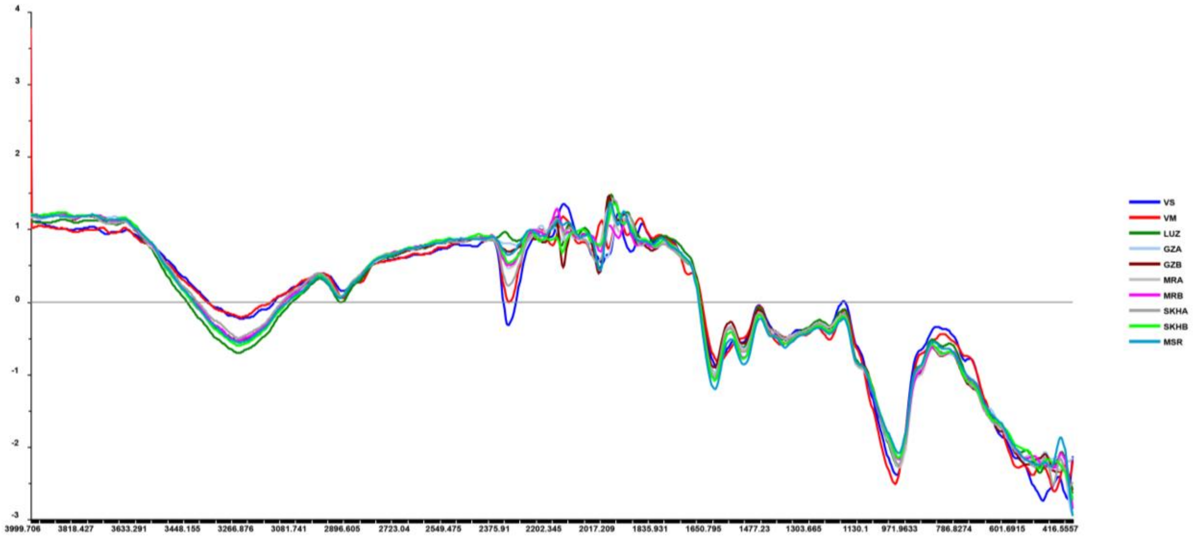

Figure 2S. The FT-IR spectra of ten different *Vicia* seed samples in the mid-IR region (4000–400 cm-) after preprocessing with Standard normal variate (SNV) algorithm to reduce the scatter effect. Each of them represents the FT-IR spectrum for one of the eight cultivars of *Vicia faba* species, as well as the two other *Vicia* species, *Vicia sativa* and *Vicia monantha*.

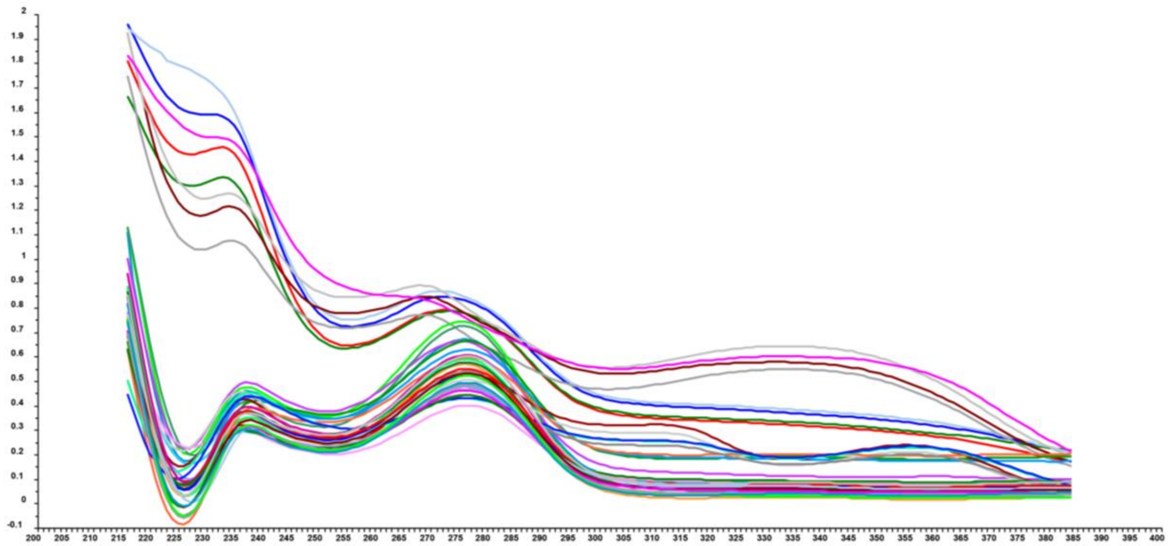

Figure 3S. The UV absorbance spectra of 40 samples of *Vicia* seeds the 200-400 nm range. The 40 samples of *Vicia* seeds are composed of four samples each of *Vicia sativa* and *Vicia monantha*, as well as 32 samples from eight fava bean varieties (4 samples per variety).

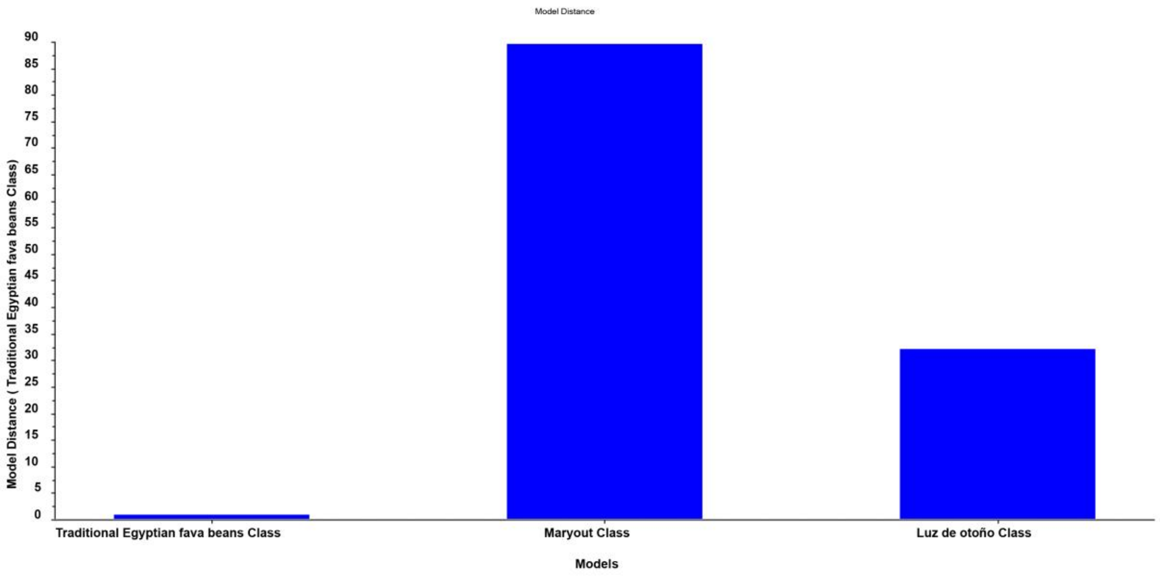

Figure 4S. Model distance relative to the traditional Egyptian fava bean class. This metric can provide information about the separation between any pair of classes. If the model distance value for certain class is greater than three, then this model is well separated from the model we compare with. In this figure, both Maryout class and Luz de otoño class are well separated from the traditional Egyptian fava bean class as they have model distance values of about 89 and 32, respectively.

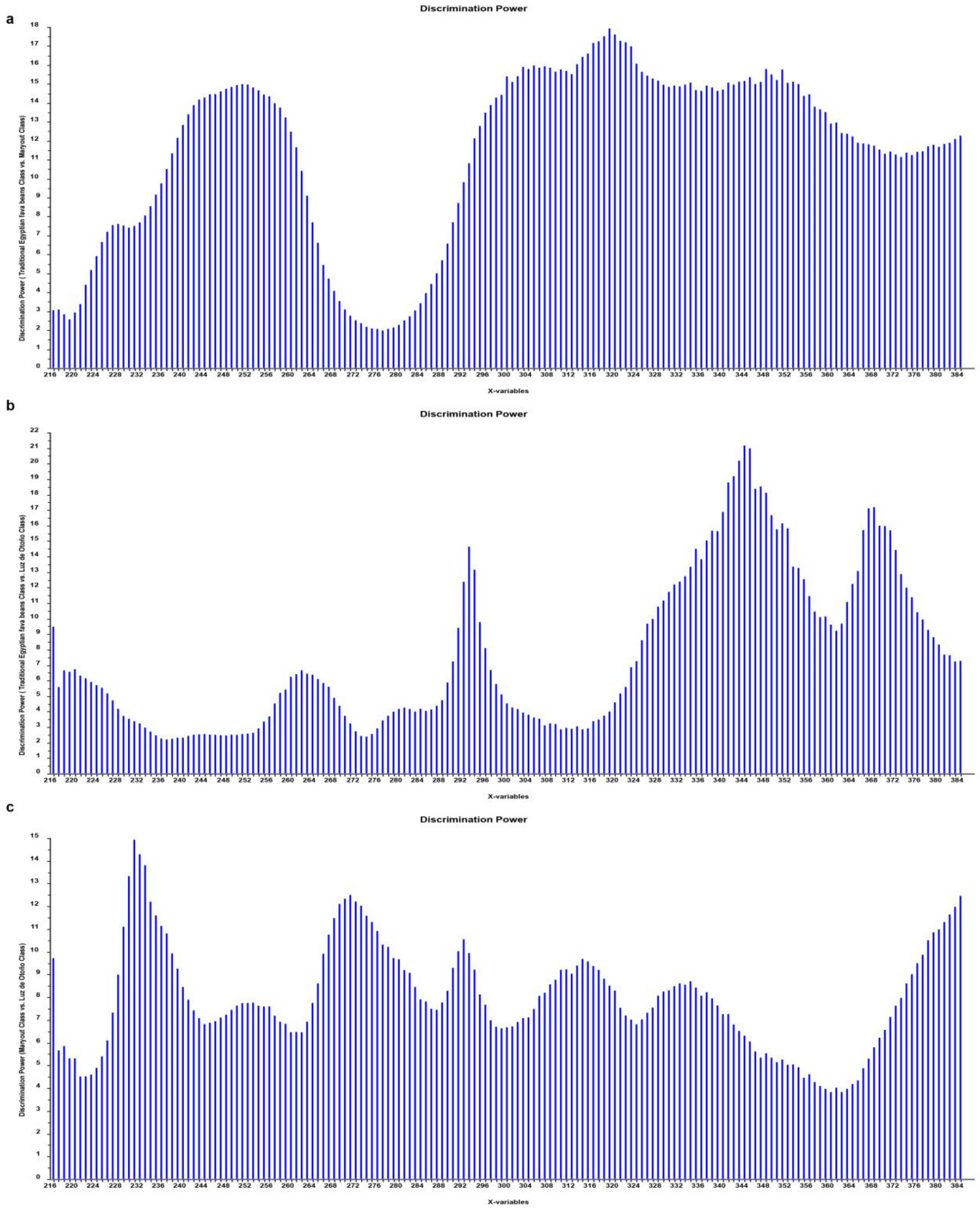

Figure 5S. The discriminatory power of spectral variables between all pairs of the three classes of fava beans. If the variable value is greater than 3, then it is important in the discrimination between these pairs of classes. The discriminatory power of spectral variables of traditional Egyptian Fava bean class vs Maryout class **(a)**, traditional Egyptian fava bean class vs Luz de otoño class **(b)**, and Maryout class vs Luz de otoño class **(c).**

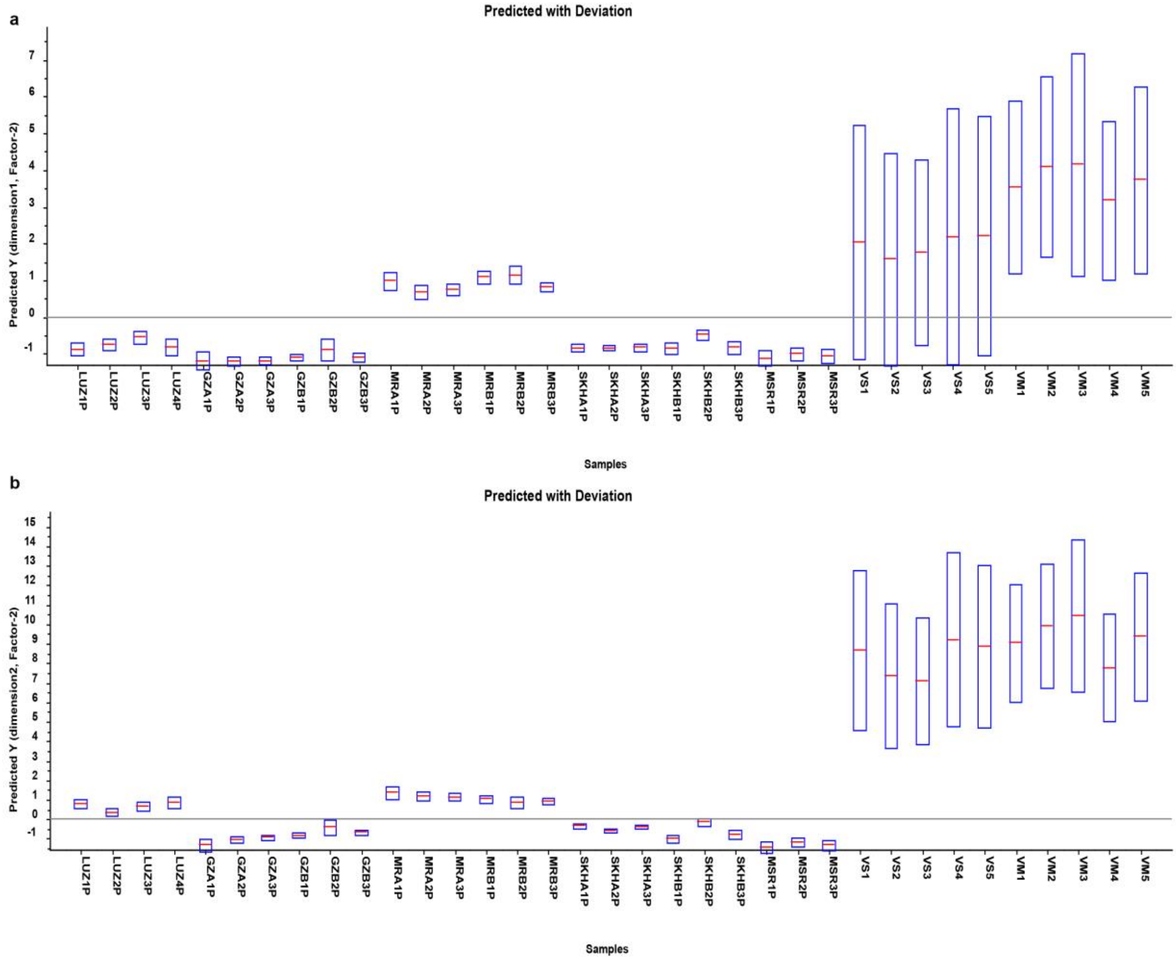

Figure 6S. showed the predicted with deviation plot from the PLS-DA discrimination of the validation set (n=35 samples), which comprises the validation samples from fava bean varieties (n=25), as well as the non-fava bean samples (n=10). Fava bean samples in the validation set were correctly classified with low deviation, whereas the *Vicia sativa* and *Vicia monantha* samples appeared as very outliers with very high deviation. Predicted Y categorical variable in the first dimension **(a**). Predicted Y categorical variable in the second dimension **(b).** To classify an unknown sample as a member of class 1 of the five Egyptian traditional fava bean varieties, the predicted Y values must be less than 0, and the deviation must be less than 1. To be considered as a member of class 2 of the Spanish variety Luz de Otoño, the predicted Y values of the first dimension must be < 0 and the second dimension must be > 0, and the deviation is < 1. To be assigned as a member of class 3 of the new Egyptian varieties (Maryout 2 and 3), both predicted Y values in the two dimensions must be greater than 0, with a deviation less than 1. The last 10 validation samples which belong to *Vicia sativa* and *Vicia monantha* have much greater deviation than 1 and so were not assigned to any of three classes of fava beans.

Table 1S. The SIMCA classification results of the validation set samples (n= 35) which comprises fava bean varieties samples (n=25) and non-fava bean samples from non- target species, *Vicia sativa* and *Vicia monantha* (n= 10). Every fava bean sample in the validation set was assigned accurately to one of the 3 classes of fava bean varieties. Furthermore, all the last 10 samples of the non-target species, *Vicia sativa* and *Vicia monantha*, were not assigned to any of the three fava bean classes.

**Sample - Class membership 5%**	**Class 1 (Traditional Egyptian Fava bean varieties)**	**Class 2 (Spanish Variety Luz de otoño)**	**Class 3 New Egyptian varieties Maryout 2 and 3**
**LUZ1P**		*	
**LUZ2P**		*	
**LUZ3P**		*	
**LUZ4P**		*	
**GZA1P**	*		
**GZA2P**	*		
**GZA3P**	*		
**GZB1P**	*		
**GZB2P**	*		
**GZB3P**	*		
**MRA1P**			*
**MRA2P**			*
**MRA3P**			*
**MRB1P**			*
**MRB2P**			*
**MRB3P**			*
**SKHA1P**	*		
**SKHA2P**	*		
**SKHA3P**	*		
**SKHB1P**	*		
**SKHB2P**	*		
**SKHB3P**	*		
**MSR1P**	*		
**MSR2P**	*		
**MSR3P**	*		
**VS1**	No	No	No
**VS2**	No	No	No
**VS3**	No	No	No
**VS4**	No	No	No
**VS5**	No	No	No
**VM1**	No	No	No
**VM2**	No	No	No
**VM3**	No	No	No
**VM4**	No	No	No
**VM5**	No	No	No
